# Identification of an Intracellular Site of Prion Conversion

**DOI:** 10.1371/journal.ppat.1000426

**Published:** 2009-05-08

**Authors:** Zrinka Marijanovic, Anna Caputo, Vincenza Campana, Chiara Zurzolo

**Affiliations:** 1 Institut Pasteur, Unité Trafic Membranaire et Pathogénèse, Paris, France; 2 Dipartimento di Biologia e Patologia Cellulare e Molecolare, Università degli Studi di Napoli ‘Federico II’, Naples, Italy; University of Edinburgh, United Kingdom

## Abstract

Prion diseases are fatal, neurodegenerative disorders in humans and animals and are characterized by the accumulation of an abnormally folded isoform of the cellular prion protein (PrP^C^), denoted PrP^Sc^, which represents the major component of infectious scrapie prions. Characterization of the mechanism of conversion of PrP^C^ into PrP^Sc^ and identification of the intracellular site where it occurs are among the most important questions in prion biology. Despite numerous efforts, both of these questions remain unsolved. We have quantitatively analyzed the distribution of PrP^C^ and PrP^Sc^ and measured PrP^Sc^ levels in different infected neuronal cell lines in which protein trafficking has been selectively impaired. Our data exclude roles for both early and late endosomes and identify the endosomal recycling compartment as the likely site of prion conversion. These findings represent a fundamental step towards understanding the cellular mechanism of prion conversion and will allow the development of new therapeutic approaches for prion diseases.

## Author Summary

The misfolded form (PrP^Sc^ or prion) of the naturally occuring prion protein (PrP^C^ or cellular PrP) is responsible for neurodegenerative diseases such as Creutzfeldt-Jakob disease (CJD), bovine spongiform encephalopathy (BSE) (also known as ‘mad cow disease’) and a new variant of CJD (vCJD), which is thought to be caused by ingestion of cattle-derived foodstuffs contaminated with prions. These diseases are characterized by the accumulation of protein deposits in the central nervous system (CNS). However, unlike other neurodegenerative diseases, prion diseases are infectious and prions are able to propagate in a chain reaction by imposing their malconformed state onto the properly folded cellular proteins. Understanding where the conversion of PrP^C^ into PrP^Sc^ occurs in cells has been an unsolved question until now. By analysing the intracellular localization of PrP^C^ and PrP^Sc^ and measuring the levels of PrP^Sc^ produced in infected neuronal cell lines under conditions in which intracellular trafficking of the protein is impaired, we found that prion conversion occurs in the endosomal recycling compartment (ERC) where it transits after being internalized from the cell surface. This study will help to clarify the cellular mechanism of the disease and it opens the way to new therapeutic strategies aimed at the conversion compartment.

## Introduction

Conversion of the cellular prion protein (PrP^C^) into a conformationally altered pathogenic form, denoted PrP scrapie (PrP^Sc^) is the central event in the pathogenesis of transmissible prion diseases [1]. In the most accredited model of prion formation and replication, a direct interaction between the pathogenic PrP^Sc^ template and the endogenous PrP^C^ substrate is proposed to drive the formation of nascent infectious prions [1]. Despite decades of research, the mechanism of prion conversion, the intracellular site where this process occurs and how it leads to neurological dysfunction remain unknown [2].

A number of studies have already attempted to identify subcellular location(s) where prion conversion occurs, mostly by analyzing PrP^C^ and PrP^Sc^ subcellular distribution and trafficking in infected cell lines [3],[4], primary neurons [5],[6],[7] and in the brains of infected animals [8],[9],[10],[11],[12] using different techniques. However, these results remain controversial and do not provide clear evidence for the involvement of any specific compartment.

PrP^C^ has been shown to localize to different compartments depending on the cell type. While Pimpinelli et al. reported a predominant localization in late endosomes of neuroblastoma-derived (N2a) and hypothalamic gonadotropin releasing (GT1-7) cell lines [13], other studies have reported that in primary neurons and in N2a cells PrP^C^ is internalized and recycled back to the cell surface, with very little being localized in lysosomes [6],[7]. Furthermore, a chimeric protein fused with GFP (GFP-PrP^C^) expressed in SN56 cells derived from septal cholinergic neurons, has been detected in the Golgi, early endosomes (EEs), and in the endosomal recycling compartment (ERC) [14]. In hippocampal neurons, PrP^C^ is found principally at the plasma membrane [5] and on vesicles resembling early endocytic or recycling vesicles [12].

Less information is available about the intracellular localization of PrP^Sc^. This is mainly due to the lack of specific antibodies and the need for protein denaturation by guanidine-hydrochloride (Gnd) in order to reveal PrP^Sc^ epitopes [15]. Earlier work reported that the majority of PrP^Sc^ is intracellular [15], sequestered within lysosomes of scrapie-infected N2a cells [3],[16],[17] with little localization at the cell surface [18]. In infected brains, PrP^Sc^ has been reported to accumulate at the plasma membrane and occasionally in late endosome/lysosome-like structures [10]. More recent reports describe accumulation of PrP^Sc^ either in the perinuclear Golgi region of neurons in scrapie-infected transgenic mice [11], in the late endosomal compartment in infected GT1-7 and N2a cells [13] and at the cell surface and on early endocytic and recycling vesicles of hippocampal neurons [12]. Furthermore exogenous Alexa-labeled PrP^Sc^ was shown to be internalized into vesicles positive for late endosomal/lysosomal markers in SN56 cells and hamster cortical neurons [14].

Several studies indicate that PrP^Sc^ is formed after PrP^C^ has reached the plasma membrane [3],[19],[20],[21]. Furthermore, the endocytic pathway has been proposed to be important for the conversion of PrP^C^ to PrP^Sc^, based on the observation that release of nascent PrP from the cell surface using phosphatidylinositol-specific phospholypase C, or inhibition of endocytosis using a temperature block prevented PrP^Sc^ synthesis [2],[3]. Moreover, PrP^Sc^ is cleaved at its N-terminus by endogenous proteases in acidic compartments immediately after its generation [3],[16], suggesting that its conversion to a protease-resistant state occurs prior to its exposure to proteases within an endo-lysosomal compartment. Furthermore, the expression of a dominant-negative version of the GTPase, Rab4a, which inhibits recycling to the plasma membrane, increases the production of PrP^Sc^ in infected N2a cells supporting the hypothesis that PrP^Sc^ formation does not require cell-surface recycling and occurs in an intracellular compartment [4]. Although no specific compartment has been identified, altogether these data provide good evidence that PrP^Sc^ may be generated either at the cell surface or more likely along the endocytic pathway.

Proteins have been shown to enter the cell through many different routes (for review see [22]; however, regardless of the internalization pathway used, cargo is first delivered to early endosomes. At this level cargo to be recycled is returned to the cell surface either by a fast-recycling pathway, directly from early endosomes, or it is transported first to the endosomal recycling compartment and then to the cell surface. In contrast, cargo destined for degradation is sorted to multivesicular late endosomes and finally to lysosomes, where degradation occurs (for review see [23],[24] and see below). Importantly, Rab GTPases, which reside in different subcellular compartments, have been identified as central regulators of intracellular transport, controlling specific fusion between vesicles and different compartments (for review see [25]). The aim of the current study was to identify the subcellular compartment(s) of PrP^Sc^ production. To this end we set up a quantitative image analysis system and monitored PrP^C^ and PrP^Sc^ localization and PK-resistant PrP levels under different experimental conditions in three different neuronal cell lines (N2a, GT1 and CAD cells) that represent established cell models for prion infection and replication [26],[27]. We show here that at the steady-state high amounts of PrP^Sc^ reside in the endosomal recycling compartment (ERC). Then by selectively perturbing PrP trafficking through the endosomal compartments we excluded roles for both early and late endosomes in prion conversion and provide evidence that this event occurs in the endosomal recycling compartment.

## Results

### Steady-state localization of PrP^C^ and PrP^Sc^ in ScGT1 cells

**Figure 1:**
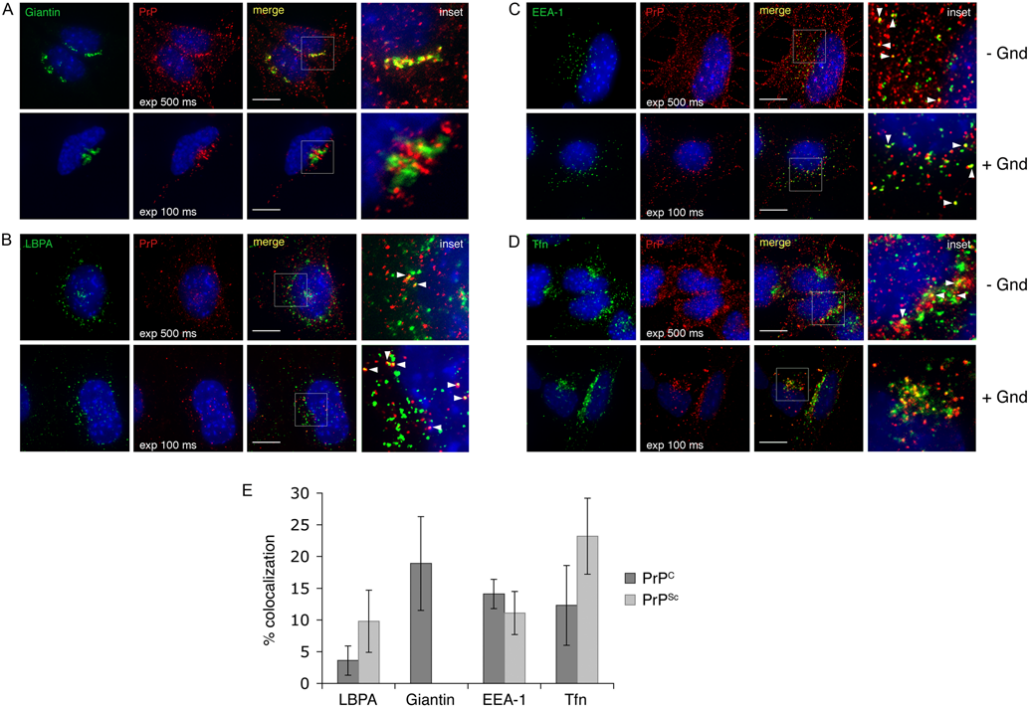
Steady state distribution of PrP^C^ and PrP^Sc^ in ScGT1 cells. PrP was immunolabeled with SAF32 mAb and colocalization with (A) Giantin (Golgi), (B) LBPA (late endosomes), (C) EEA-1 (early endosomes) and (D) Alexa 488-transferrin (Tfn, marker for the endosomal recycling compartment) was analyzed. Exp 500 ms and exp 100 ms represent the exposure times used to detect immunofluorescent signal coming from PrP^C^ and PrP^Sc^ respectively. Yellow color and arrowheads indicate colocalization. Nuclei are stained with DAPI (blue). Insets represent magnifications of the boxed areas. Scale bars represent 10 µm. (E) Quantification of PrP^C^ and PrP^Sc^ colocalization with LBPA, Giantin, EEA-1 and Alexa 488-transferrin.

Because the subcellular distribution of PrP forms may yield clues as to the site of prion conversion, we quantitatively analyzed the intracellular distribution of PrP^C^ and PrP^Sc^ in infected GT1 cells (ScGT1) by using specific antibodies and a high resolution wide-field microscope (Marianas, Intelligent Imaging Innovations) together with different imaging software packages (see Methods). Based on colocalization with different organelle markers, we found ∼20% of PrP^C^ localized in the Golgi (Figure 1A, upper panels and Figure 1E), ∼15% in early endosomes (EEs) (Figure 1C, upper panels and Figure 1E), ∼15% in the endosomal recycling compartment (ERC) (Figure 1D, upper panels and Figure 1E) and only ∼3% in late endosomes (LEs) (Figure 1B, upper panels and Figure 1E). The majority of the protein (∼50%) was localized at the cell surface (data not shown). Interestingly, we observed the same distribution of PrP^C^ in uninfected GT1 cells, indicating that PrP^Sc^ infection did not alter PrP^C^ trafficking in GT1 cells (data not shown). Interestingly, in two other infected neuronal cell lines, ScCAD and ScN2a, PrP^C^ was almost exclusively localized at the cell surface (data not shown).

Due to the lack of PrP^Sc^ - specific antibodies [15] in order to visualize PrP^Sc^, a denaturation step with guanidine hydrochloride (Gnd) is required. However, this treatment does not allow one to easily distinguish PrP^Sc^ from PrP^C^. In contrast to previous studies, we took advantage of the features of our imaging system to discriminate PrP^Sc^ from PrP^C^ by adjusting the signal threshold, and recording only the higher signal intensities characteristic of PrP^Sc^ staining after Gnd treatment in infected cells (see Methods, Figure S1 and [28]). Because we were concerned that with this stringent approach we would detect mainly the brighter PrP^Sc^ signal which could derive from large aggregates, we performed velocity gradients in order to analyze the state of PrP aggregation, similar to what has been shown before in brain [29]. Interestingly, we did not observe any difference in the distribution of PrP on the gradients between uninfected cells containing only PrP^C^ and infected cells containing both PrP^C^ and PrP^Sc^, thus arguing against the presence of large PrP^Sc^ aggregates in our cell model (data not shown) in contrast to what was described before in infected brains [29],[30]. These data, although indirectly, support the use of this thresholding procedure to reveal (with a reasonable approximation) the intracellular distribution of all PrP^Sc^ in infected cells as recently shown [28],[31]. By this approach we observed that unlike PrP^C^, only ∼10% of PrP^Sc^ was localized at the cell surface (data not shown), while ∼90% was found to be intracellular as previously described [15],[18]. However, in contrast to earlier reports [11],[13],[17] we did not observe any localization of PrP^Sc^ in the Golgi compartment (Figure 1A lower panel) the majority of the intracellular protein being localized throughout the endocytic pathway. While only ∼10% of the protein was localized in LEs (Figure 1B lower panels and Figure 1E) and EEs, (Figure 1C lower panels and Figure 1E) more than 25% was found in the ERC (Figure 1D lower panels and Figure 1E). Interestingly, a similar distribution of PrP^Sc^ was observed in ScCAD and ScN2a cells (Figure S2). Therefore, the greater amount of PrP^Sc^ in the ERC when compared to other subcellular sites could indicate a potential involvement of this compartment in the conversion process. Alternatively, the ERC could just represent the compartment through which PrP^Sc^ recycles after being converted elsewhere (eg. at the cell surface, in EEs or in LEs, which have previously been proposed as sites of conversion) [17],[32],[33]. To directly examine this question we selectively perturbed the trafficking through the different endosomal compartments using pharmacological inhibitors or mutant proteins affecting the different pathways.

### Impaired exit from early endosomes reduces PrP^Sc^ levels

**Figure 2:**
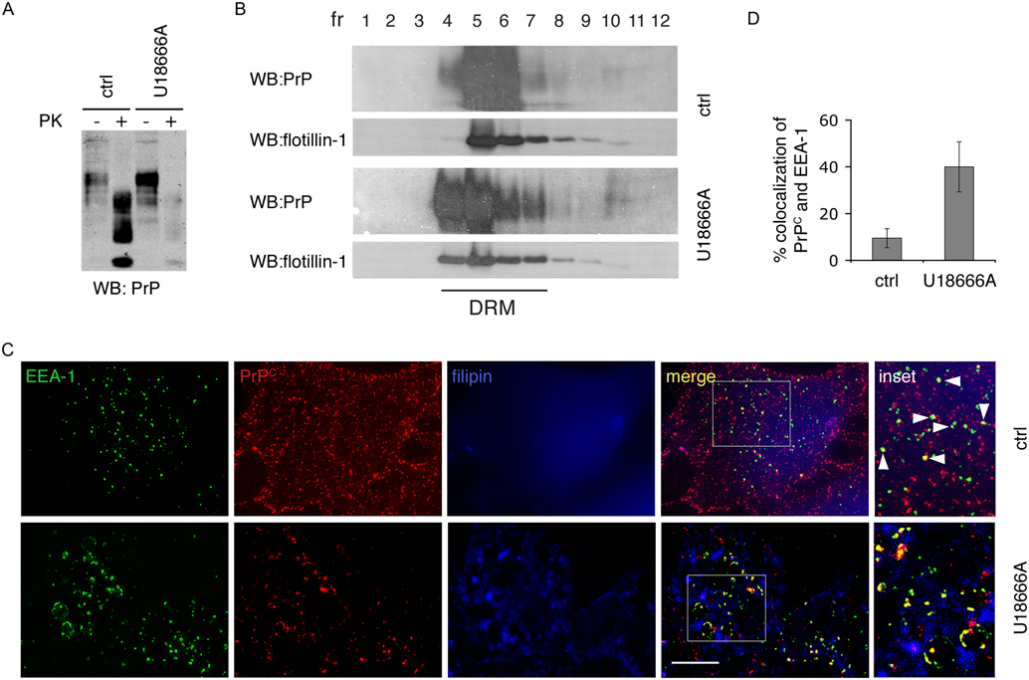
U18666A reduces PrP^Sc^ levels and impairs PrP^C^ trafficking in ScGT1 cells, without affecting PrP distribution in DRMs. (A) Levels of total PrP (PK−) or PrP^Sc^ (PK+) were analyzed on western blot in control cells (ctrl) and cells treated with U18666A. Increased PrP^C^ and decreased PrP^Sc^ levels were observed in treated cells. (B) Lysates from control and U18666A treated ScGT1 cells were applied on sucrose gradients and ultracentrifuged at 200000 g for 16 hr. Twelve fractions were collected and proteins were precipitated using 10% of trichloroacetic acid. PrP and flotillin-1 contents in each fraction were analyzed on western blot using SAF61 mAb and flotillin-1 mAb. Fractions 4–7 correspond to detergent resistant membranes (DRM) based on distribution of flotillin-1, which is mainly present in DRMs. In ScGT1 cells PrP is distributed in DRMs and U18666A treatment does not change its distribution. (C) Steady-state localization of PrP^C^ was analyzed in control (ctrl) and U18666A-treated ScGT1 cells. Yellow color and arrowheads represent colocalization between PrP^C^ and EEA-1. Scale bars represent 10 µm (D) Quantification results are presented as % (mean±s.e.m, n = 80) of total PrP^C^ signal (red) colocalizing with EEA-1 signal (green) (p = 0,014, t-test).

To examine the role of the late endocytic pathway in prion conversion, we treated ScGT1 cells with U18666A. This compound triggers cholesterol accumulation in LEs and lysosomes by an as of yet unknown mechanism [34]. As a consequence, it effectively inhibits trafficking from EEs to LEs, because Annexin II, which is coordinating this step is redistributed into cholesterol laden LEs. In addition, exit from LEs to the Golgi and lysosomes is inhibited and protein degradation is impaired [35],[36]. After treating ScGT1 cells with U18666A for 6 days, we observed a complete disappearance of Proteinase K (PK) resistant PrP^Sc^, as shown by limited proteolysis using the PK assay (Figure 2A and see the explanation in Methods). We also observed an increase in total PrP^C^ levels as expected because of the known effect of U18666A on protein degradation [35] (Figure 2A). Therefore, under these conditions the reduction in PrP^Sc^ was likely due to inhibition of PrP^Sc^ production rather than its increased degradation. Interestingly, PrP^Sc^ reduction following U18666A treatment was previously reported in ScN2a cells and was attributed to the redistribution of PrP^C^ outside of cholesterol and glycosphingolipids enriched membrane microdomains, known as detergent-resistant domains (DRMs) or lipid rafts [37]. This is conceivable considering the effect of this drug on cholesterol and sphingolipid trafficking [38]. However, although we could reproduce the results in ScN2a cells (Figure S3A), we did not observe any effect of U18666A on the association of PrP^C^ with DRMs neither in ScN2a (Figure S3B) nor in ScGT1 cells (Figure 2B), thus refuting the above hypothesis. In contrast, we observed that in both cell lines U18666A treatment altered the subcellular distribution of PrP^C^, which became highly enriched (∼40%) in EEs after the treatment (compare control cells with treated cells in Figure 2C and Figure S3C, and see quantification of EEA-1/PrP colocalization in Figure 2D). Therefore, these data suggest that U18666A affects PrP conversion by altering its intracellular trafficking. Furthermore, they also indicate that PrP^C^ must exit the EE compartment in order to be converted to PrP^Sc^.

**Figure 3:**
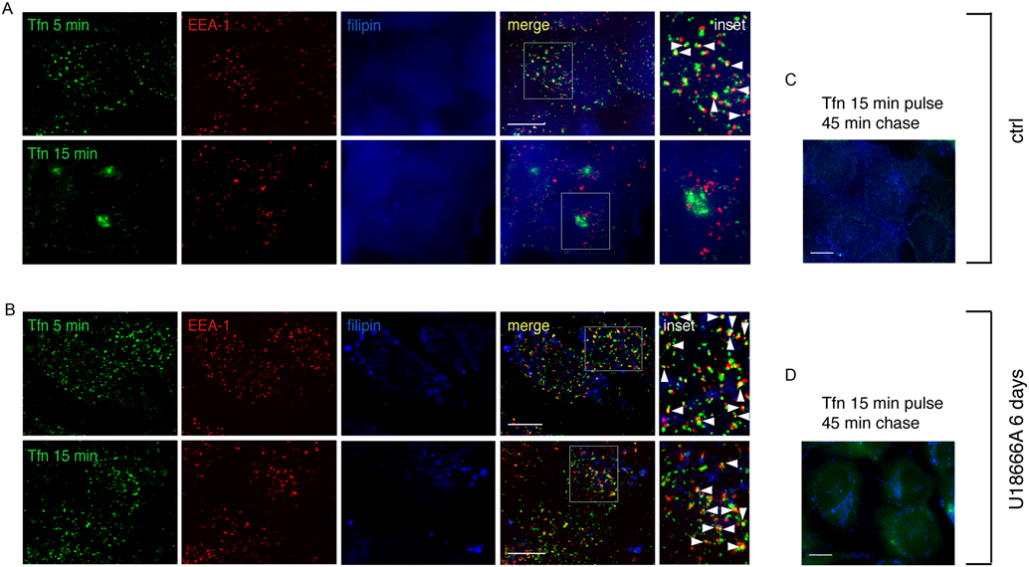
U18666A blocks transferrin trafficking from EE to ERC but does not interfere with Tfn delivery to the cell surface. (A) Recycling of Alexa 488-transferrin (Tfn) was analyzed in control ScGT1 cells. A diffused cholesterol distribution was revealed by filipin staining (blue). Yellow color and arrowheads indicate colocalization between Tfn and EEA-1. Inset represents magnification of the boxed area. (B) The same experiment described in (A) was performed in ScGT1 cells treated with U18666A for 6 days. Characteristic large cholesterol laden endosomes were revealed by fillipin staining (blue). Note that in contrast to control cells, in treated cells Tfn was not transported to ERC 15 min post-internalization. Alexa 488-transferrin was chased out of both control (C) and U18666A (D) treated cells after 45 min. Scale bars represent 10 µm.

To identify the pathway involved in prion conversion we had to determine to what extent the U18666A treatment affected endocytic pathways in ScGT1 cells. To this aim we analysed the trafficking of two molecules widely used to characterize trafficking through endocytic compartments: dextran, which traffics through EEs and reaches LEs, and transferrin (Tfn) which recycles back to the surface through EEs and the ERC. By following fluid phase uptake of fluorescently tagged dextran (Figure S4) we confirmed that U18666A also inhibits the traffic from EEs to LEs in ScGT1 cells (Figure S4B) as previously reported for other cell types [35],[36]. Furthermore, by staining cholesterol with filipin, we also show that U18666A causes enlargement of LEs, which accumulate cholesterol (see large organelles in Figure S4B, costained with filipin and LBPA). In addition to this well documented effect, we found that in U18666A treated cells fluorescently tagged Tfn was not able to reach the ERC and after 15 minutes of internalization was still arrested in EEs both in ScGT1 (Figure 3B) and ScN2a cells (Figure S3D). Nonetheless, we observed that, similar to control ScGT1 cells, Tfn could not be detected inside the cells after a 45 minutes chase period (compare panels c and d in Figure 3), indicating that under these conditions Tfn recycling to the surface via EEs was unaffected. These data therefore show that prolonged treatment with U18666A did not alter arrival to EEs nor recycling from EEs to the PM but inhibited trafficking pathways both between EEs and LEs and between EEs and the ERC. Since endogenous PrP^C^ accumulates in EEs in the presence of U18666A (Figures 2C and 2D), overall these results indicate that EEs (and recycling from EEs to the PM) are not involved in PrP^Sc^ production, while exit of PrP from EEs towards either the ERC or LEs is required for conversion to occur. Therefore we decided to selectively inhibit these two pathways and to analyze the effect on scrapie production.

### Late endosomes are not involved in prion conversion

**Figure 4:**
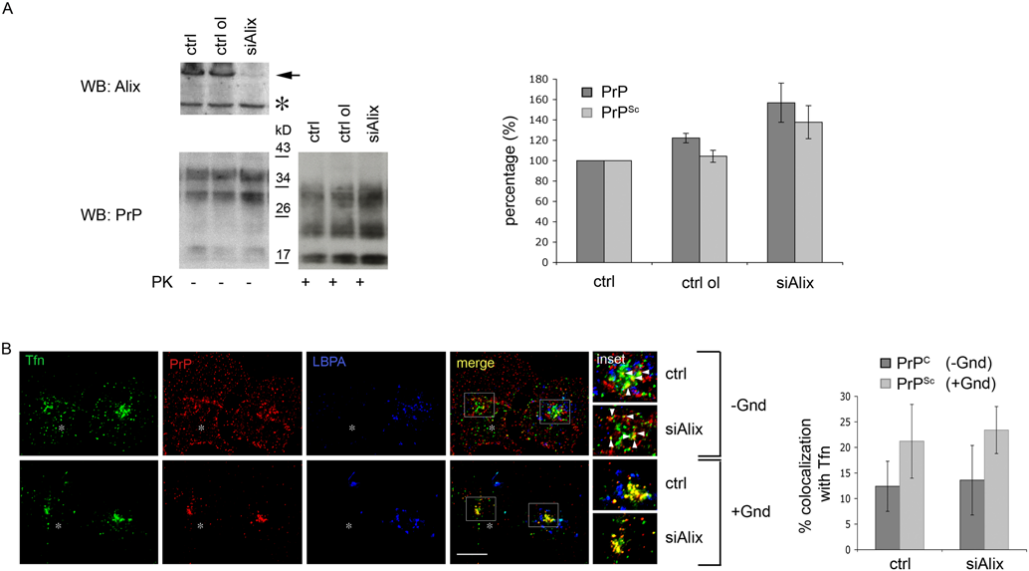
Alix downregulation in ScGT1 cells does not affect PrP localization and PrP^Sc^ levels. (A) Alix, PrP^Sc^ (PK+) and total PrP (PK−) levels were analyzed on western blot in control (ctrl) cells and cells transfected either with control oligo (ctrl ol) or RNAi against Alix (siAlix). The band corresponding to Alix is marked by an arrow. Asterisk indicates unspecific band as a control for equal loading. Slightly increased levels for total PrP (p = 0,038) and PrP^Sc^ (p = 0,018) were observed upon Alix downregulation. (B) Steady state distribution of PrP^C^ (−Gnd) and PrP^Sc^ (+Gnd) in control (ctrl) and Alix depleted cells (siAlix) was analyzed by immunofluorescence. Alix depleted cells containing significantly less LBPA positive vesicles (blue) are marked by asterisk. Yellow color and arrowheads indicate colocalization between PrP and Tfn. The quantification results (mean±s.e.m, n = 80) are presented as % of total PrP signal (red) colocalizing with Tfn (green). Note that no difference was observed in PrP^C^ (p = 0,83, t-test) and PrP^Sc^ (p = 0,52, t-test) colocalization with transferrin in control condition and upon Alix downregulation. Scale bars represent 10 µm.

In order to directly test whether LEs/lysosomes are involved in PrP^Sc^ production, we used an RNAi approach to downregulate Alix, a protein that is required for the biogenesis of LEs [39] (Figure S5A). We obtained a clear reduction of protein levels (∼80%) after transfection of Alix siRNA in ScGT1 and ScCAD cells (Figure 4A and Figure S5B), but not in ScN2a cells (data not shown). Consistent with previous findings in HeLa cells [39] the number of LEs and lysosomes in transfected ScGT1 cells was drastically reduced (∼10 fold) (compare top and bottom panels in Figure S5A and quantifications). However, despite the drastic reduction in the number of LEs, the levels of total PrP and PrP^Sc^ were unaltered in Alix-depleted ScGT1 cells. Instead, we observed a slight increase in PrP^Sc^ as well as total PrP, likely due to the reduction in protein degradation in cells with fewer LEs/lysosomes (Figure 4A). Interestingly, we could also reproduce these data in ScCAD cells (Figure S5B), indicating that the effect of Alix depletion on PrP^Sc^ was not limited to a single infected cell line. Overall, these data demonstrate that PrP transport to LEs is not required for PrP^Sc^ production and rule out the involvement of LEs in scrapie conversion. Interestingly, we observed that Alix depletion did not affect the intracellular localization of PrP^C^ and PrP^Sc^, which were found to be enriched in the ERC similar to control cells (Figure 4B, compare colocalization with Tfn in control and treated cells for PrP^C^ (−Gnd) and PrP^Sc^ (+Gnd) and see quantification in the right panel), thus, pointing to a role for this compartment in prion conversion.

### Impaired recycling from early endosomes does not affect PrP^Sc^ levels

In U18666A-treated cells Tfn delivery to the ERC was inhibited (Figure 3B) but its recycling to the surface from EEs was unaffected (Figures 3C and 3D). Under the same conditions, PrP was enriched in EEs (Figure 2B). These data suggest that ERC-independent recycling to the surface *via* EEs is not likely to be relevant for PrP^Sc^ production. Consistent with this hypothesis expression of a dominant-negative Rab4 mutant (GFP-Rab4N121I), which impairs recycling from EEs [40], did not affect PrP^Sc^ levels in either ScGT1 or ScN2a cells (Figure S6).

Interestingly, it was previously reported that in ScN2a cells the same Rab4 dominant-negative mutant increased scrapie levels [4]. Although in our hands the levels of PrP^Sc^ remained unaltered in ScN2a cells transfected with Rab4 dominant-negative, both of these sets of data concur in showing that recycling from EEs to the PM is not involved in scrapie production.

### PrP sorting to the endosomal recycling compartment is required for PrP^Sc^ production

**Figure 5:**
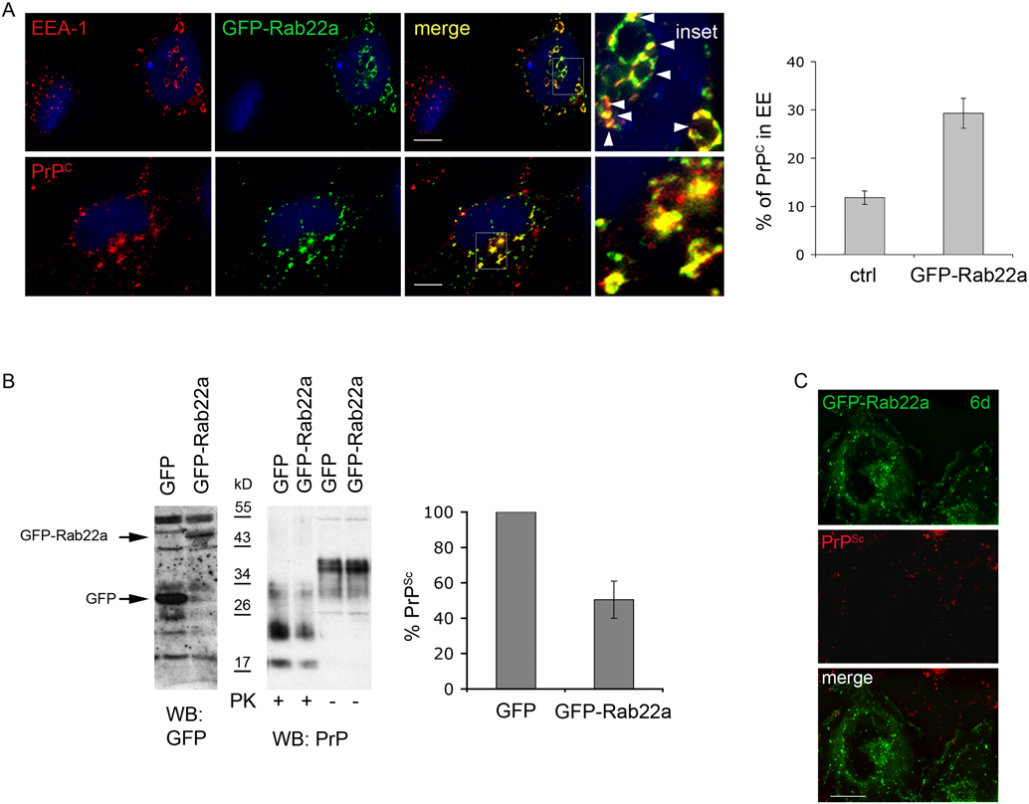
Overexpression of GFP-Rab22a affects cellular distribution of PrP^C^ and reduces PrP^Sc^ levels. (A) ScGT1 cells were transfected with GFP-Rab22a and immunolabeled for EEA-1 or PrP^C^. Yellow color and arrowheads indicate colocalization. The quantification results (mean±s.e.m, n = 36) are presented as % of total signal in red (PrP) colocalizing with GFP-Rab22a (green). Increase in PrP^C^ (p = 0,0011, t-test) in EE of GFP-Rab22a expressing cells was detected. (B) GFP and GFP-Rab22a in transfected ScGT1 cells were analyzed on western blot using anti-GFP Abs. Band corresponding to GFP and GFP-Rab22a are marked with arrows. Levels of PrP^Sc^ (PK+) and total PrP (PK−) were analyzed on western blot. The PrP^Sc^ level (mean±s.e.m, n = 3) in GFP-Rab22a expressing cells is presented as % of the PrP^Sc^ level in GFP expressing cells, which is considered as 100%. Overexpression of GFP-Rab22a in ScGT1 cells causes reduction in PrP^Sc^ levels (p = 0,015, t-test). (C) ScGT1 cells were transfected with GFP-Rab22a (green) for 6 days and immunolabeled for PrP^Sc^ upon Gnd treatment using Saf32 mAb. Note that in contrast to control cells, PrP^Sc^ (red) cannot be observed in GFP-Rab22a expressing cells. Scale bars represent 10 µm.

To directly test whether PrP^C^ must reach the ERC in order to be converted to PrP^Sc^ we analyzed the effect of Rab22a overexpression on PrP sorting and PrP^Sc^ production. Rab22a has been shown to regulate Tfn sorting from EEs to the ERC [41] and to be involved in sorting of MHCI from the ERC into tubular carriers destined for the cell surface [42]. In CHO cells overexpression of GFP-Rab22a causes a characteristic enlargement of EEs [41], probably due to enhanced homotypic fusion of Rab22-containing vesicles. This prevents segregation of the domains required for fusion with other compartments, resulting in delayed transport out of EEs. As a consequence, Tfn accumulates in this compartment [41]. Importantly, Rab22 overexpression in CHO cells does not impair protein delivery from EEs to LEs. In contrast to CHO cells, when GFP-Rab22a is overexpressed in HeLa cells it localized to the ERC and to tubular carriers and does not affect either the distribution or the recycling of Tfn [42]. In order to see whether in our infected cell models Rab22a was acting as a specific effector of trafficking between EEs and the ERC, we analyzed the subcellular distribution of GFP-Rab22a and the effect of its overexpression on Tfn trafficking in our three infected cell models. Similar to CHO cells, in transfected ScGT1 cells we observed enlarged EEA-1 positive EEs decorated with GFP-Rab22a (Figure 5A). We also found that the transfected cells were able to internalize Tfn normally (Figure S7A). However, in contrast to control cells, Tfn was not transported to the ERC after internalization, but remained inside EEs (∼80%) (Figure S7A). Similarly PrP was enriched in the EEs of Rab22a-transfected ScGT1 cells (Figure 5A) as shown by quantification of its co-localization with EEA-1 (see graph in Figure 5A). These data therefore indicate that, like Tfn, PrP was not able to reach the ERC in Rab22a-transfected ScGT1 cells. Interestingly, by performing PK assay we observed that while total PrP levels were unchanged, there was ∼50% decrease in PrP^Sc^ levels in GFP-Rab22a-expressing cells compared to control cells transfected with GFP alone (Figure 5B, middle and right panels). This partial decrease in PrP^Sc^ levels corresponded to the transfection efficiency, which was approximately 50% (data not shown). In agreement with the biochemical data, when GFP-Rab22a transfected cells were analyzed by immunofluorescence after Gnd treatment, no PrP^Sc^ signal could be observed in GFP-Rab22a-expressing cells (Figure 5C). Interestingly, like in ScGT1 cells, GFP-Rab22a was mainly localized in enlarged EEA-1 positive endosomes in transfected ScCAD cells (Figure S8A, upper panels). However, a substantial amount of GFP-Rab22a was also found in perinuclear tubular structures (Figure S8A upper panels, arrowheads) and vesicles lacking EEA-1 (Figure S8A upper panels, arrows). Despite this heterogenous distribution, sorting of Tfn from EEs to the ERC was impaired in GFP-Rab22a expressing CAD cells similar to ScGT1 cells (Figure S8A lower panels). Therefore, as expected, Rab22a expression also caused redistribution of PrP^C^ to EE and decreased PrP^Sc^ production in this cell line similar to what was observed in ScGT1 cells (Figures S8B, S8C and S8D). In contrast, in ScN2a cells, Rab22a behaved similarly to HeLa cells and did not inhibit the trafficking of Tfn from EEs to the ERC. Consequently, overexpression of Rab22a in ScN2a cells did not have any effect on the levels of PrP^Sc^ (data not shown). Although these data indicate that PrP^C^ must reach the ERC in order to be converted, we had to rule out the possiblity that overexpressed Rab22a was titrating out other cellular sorting factors. To this end, we used a complementary approach and downregulated endogenous Rab22a using siRNA. Given the role of Rab22 in cargo sorting towards the ERC [41], depletion of Rab22a should cause a delay in transport from EEs, resulting in the same effect as Rab22 overexpression. As expected, when endogenous Rab22a was depleted from ScGT1 cells a similar decrease in PrP^Sc^ levels was obtained (Figure S7B).

**Figure 6:**
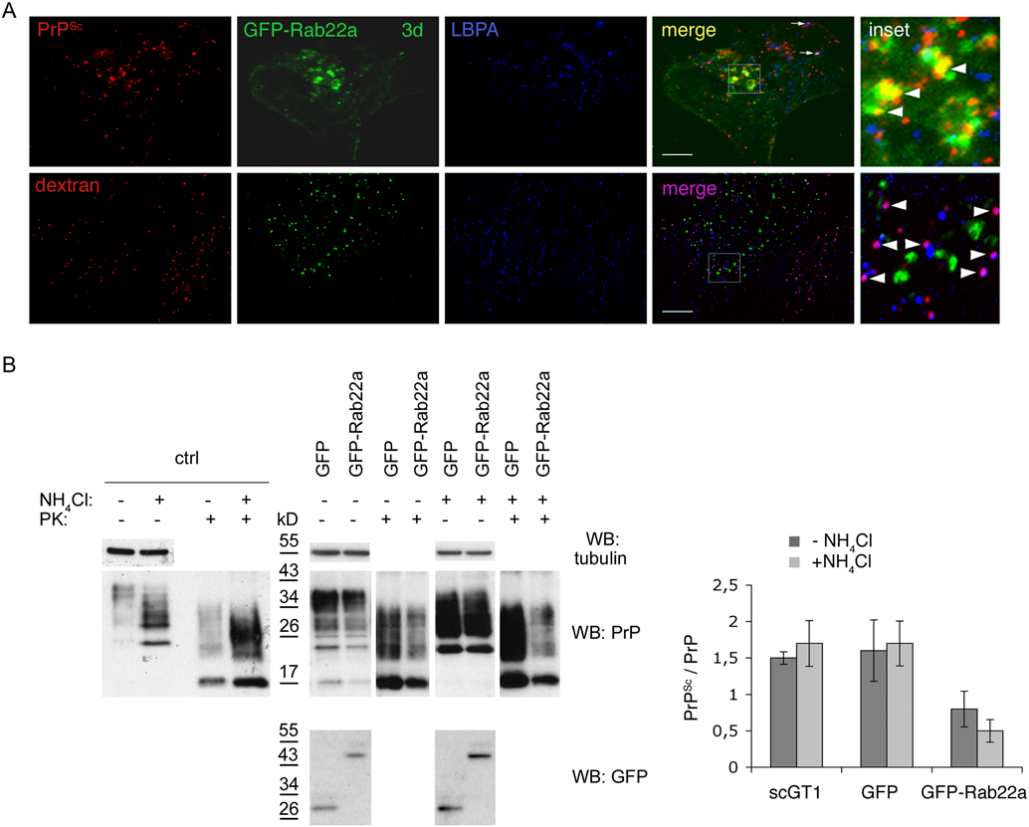
Overexpression of GFP-Rab22a does not induce PrP^Sc^ degradation. (A) GFP-Rab22a was expressed in ScGT1 cells for 3 days and PrP^Sc^ and Alexa 546-dextran distribution was analyzed by immunofluorescence. Yellow color and arrowheads indicate colocalization between PrP^Sc^ and GFP-Rab22a, while purple color indicates colocalization between dextran and LBPA (arrowheads) or PrP^Sc^ and LBPA (arrows). Scale bars represent 10 µm. (B) Control ScGT1 (ctrl) and ScGT1 transfected with GFP or GFP-Rab22 were either treated (+) or not (−) [13] with NH_4_Cl. Levels of GFP, GFP-Rab22a, PrP^Sc^ (PK+) and total PrP (PK−) were analyzed on western blot. Tubulin levels represent control for equal loading. PrP^Sc^ levels were quantified and plotted as a ratio between PrP^Sc^ and total PrP. Note that NH_4_Cl does not affect Rab22a-induced decrease in PrP^Sc^ levels (p = 0,083, t-test).

Altogether, these data indicate that a block of PrP^C^ trafficking from EEs to the ERC reduces levels of PrP^Sc^ in infected cells of different neuronal origin. Nonetheless, they do not allow us to discriminate whether the reduction in PrP^Sc^ levels is due to impaired production or to enhanced degradation of PrP^Sc^. In particular, it is possible that impairment of PrP recycling in GFP-Rab22a expressing cells could divert PrP towards a degradation pathway. To rule out this hypothesis we monitored the distribution of PrP^Sc^ and Alexa-546 dextran in ScGT1 cells expressing GFP-Rab22a three days post-transfection, when the reduction of PrP^Sc^ was not complete and when we could still detect PrP^Sc^ in the transfected cells. Under these conditions dextran was mainly found in LEs after the 3 h chase period (Figure 6A, lower panels), confirming that Rab22a did not affect the routing of dextran from EEs to LEs [41]. Conversely, only a minority of PrP^Sc^ was localized in LE under these conditions similar to control cells (Figure 6A, upper panels, arrows). Therefore, these data indicate that in GFP-Rab22a-expressing cells PrP was not diverted toward LEs. Instead, a significant amount of PrP^Sc^ was distributed in GFP-Rab22a-positive EEs, similar to what was observed for PrP^C^ (Figure 6A, upper panels, arrowheads and Figure 5A). Altogether, these results indirectly suggest that the observed decrease in PrP^Sc^ levels in Rab22-expressing cells is not due to increased delivery and degradation of PrP^Sc^ in LEs, but rather is due to impaired scrapie production. To further test this hypothesis and to discriminate between increased degradation and decreased production of PrP^Sc^, we compared the levels of PrP^Sc^ in control cells and in cells transfected with GFP-Rab22a after treating them with ammonium chloride (NH_4_Cl), which impairs lysosomal degradation [43]. We reasoned that if the reduction of PrP^Sc^ by Rab22a was due to increased lysosomal degradation, a block of the lysosomal function with NH_4_Cl should revert its effect and result in unchanged or increased PrP^Sc^ levels. On the the other hand, if the decrease of PrP^Sc^ levels by Rab22a-expression was not due to increased PrP^Sc^ degradation but rather to inhibition of PrP^Sc^ synthesis, NH_4_Cl treatment should not interfere and we should still observe a decrease in PrP^Sc^ levels. As expected from a block of lysosomal degradation we observed an increase in total cellular PrP levels in NH_4_Cl-treated cells (in both control cells transfected with GFP and in cells transfected with GFP-Rab22a) when compared to untreated cells (Figure 6B right panels). Similarly, PrP^Sc^ levels were also increased in control cells (expressing GFP alone) treated with NH_4_Cl (Figure 6B right panels). However, in contrast to total PrP, PrP^Sc^ levels were reduced in GFP-Rab22a expressing cells, independently of the presence of NH_4_Cl (Figure 6B right panels). These results therefore suggest that the observed reduction of PrP^Sc^ levels in GFP-Rab22a expressing cells is due to impaired PrP^Sc^ production rather than to increased degradation.

### The Endosomal recycling compartment is the likely site of prion conversion

**Figure 7:**
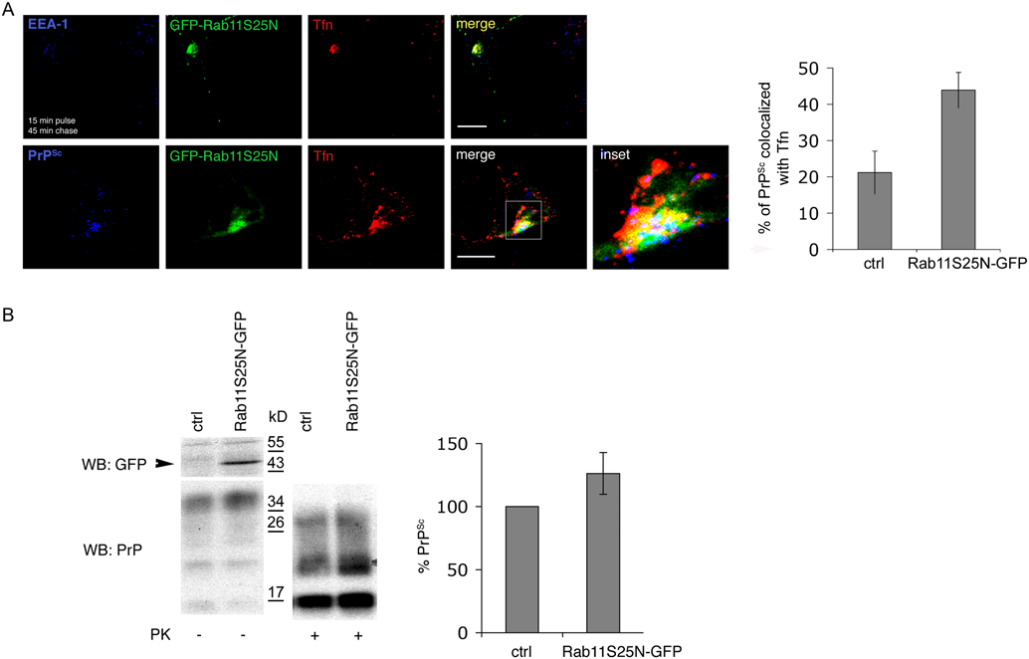
Overexpression of Rab11S25N-GFP impairs recycling of transferrin and PrP^Sc^ from the ERC and increases PrP^Sc^ production. (A) Distribution of Alexa 546-transferrin (Tfn) and PrP^Sc^ was analyzed in ScGT1 cells transfected with Rab11S25N-GFP for 3 days. Yellow color shows colocalization between Rab11S25N-GFP and Tfn, magenta between Tfn and PrP^Sc^, cyan between Rab11S25N-GFP and PrP^Sc^ and white between all three proteins. Scale bars represent 10 µm. The quantification results (mean±s.e.m, n = 45) are presented as % of total signal in blue (PrP^Sc^) colocalizing with Tfn (red) in untransfected or Rab11S25N-GFP expressing (green) cells (p = 0,0014, t-test). (B) Levels of Rab11S25N-GFP, total PrP and PrP^Sc^ were analyzed on western blot in control (ctrl) and transfected cells (Rab11S25N-GFP). The band corresponding to Rab11S25N-GFP is marked by an arrow. The quantification results (mean±s.e.m. n = 4) are plotted as percentage (%) of PrP^Sc^ levels in Rab11S25N-GFP transfected cells in comparison to untransfected (ctrl) cells where PrP^Sc^ levels are considered to be 100% (p = 0,03, t-test).

Overall, the results described in the previous paragraph indicate that the recycling of PrP through the ERC is required for scrapie production. However, they do not allow us to discriminate whether PrP^C^ to PrP^Sc^ conversion occurs within this compartment or upon return to the cell surface. In order to directly analyze the involvement of the ERC in prion conversion, we overexpressed in ScGT1 cells wild type and dominant-negative forms of Rab11, which have been shown to modulate recycling through ERC. Similar to what has been shown in other cell lines [44],[45], we found both Rab11wt-GFP and Rab11S25N-GFP in the Golgi, in the ERC, and occasionally in peripheral vesicular structures that were negative for EEA-1 (Figure S9 and data not shown). In contrast to previous reports in CHO and BHK cells [44], overexpression of Rab11S25N-GFP in ScGT1 cells did not interfere with the transport of Tfn from EEs to the ERC (Figure S9) but impaired its recycling from the ERC to the PM (Figure 7A). Indeed, in contrast to control cells in which the majority of Tfn was chased out of the cells after 45 min, in cells overexpressing Rab11S25N-GFP exit of Tfn from the ERC was delayed. While a significant amount of the protein was still detected inside the cells after a 45 min chase period (Figure 7A upper panels), complete clearance of Tfn occurred only after 90 min (data not shown). Interestingly, compared to control cells, a higher amount of PrP^Sc^ was observed to colocalize with Tfn in the ERC of transfected cells (Figure 7A lower panels) indicating that PrP was also retained in this compartment. Significantly, in these cells the levels of PrP^Sc^ were slightly increased (Figure 7B). We also attempted to analyze PrP^Sc^ levels in ScCAD and ScN2a cells expressing Rab11S25N-GFP. However, due to increased mortality of Rab11S25N-GFP-expressing cells we were not able to perform a quantitative analysis of PK-resistant PrP levels. Nonetheless, immunofluorescence analysis in the transfected cells revealed the presence of PrP^Sc^ in these cells (data not shown), thus supporting the result obtained in ScGT1 cells. Overall these data strongly suggest that PrP^Sc^ conversion occurs in the ERC and imply that recycling from ERC to the PM is not relevant to this process.

## Discussion

Conversion of PrP^C^ to PrP^Sc^ is the key event in prion pathogenesis. Prion conversion is thought to occur at a site where the two protein forms meet and are allowed to physically interact. To date there is no direct evidence for the involvement of any specific intracellular compartment in this event as several compartments have been proposed to have a role in different cell systems [11],[12],[13],[16],[17],[32]. Based on analyses of the subcellular localization of different PrP forms, lysosomes have been proposed as a possible location for the conversion process [13],[17],[32]. However, contrasting data about PrP^C^ and PrP^Sc^ localization exist [6],[7] (and see introduction), underlying the need for a more systematic and quantitative approach aimed at uncovering the site of conversion. The inconsistencies in the reported localization of different PrP forms are most likely related to the lack of PrP^Sc^ antibodies that can distinguish between the two prion forms and the need for protein denaturation by guanidine hydrochloride (Gnd) to reveal PrP^Sc^ epitopes making such analyses quite difficult.

**Figure 8:**
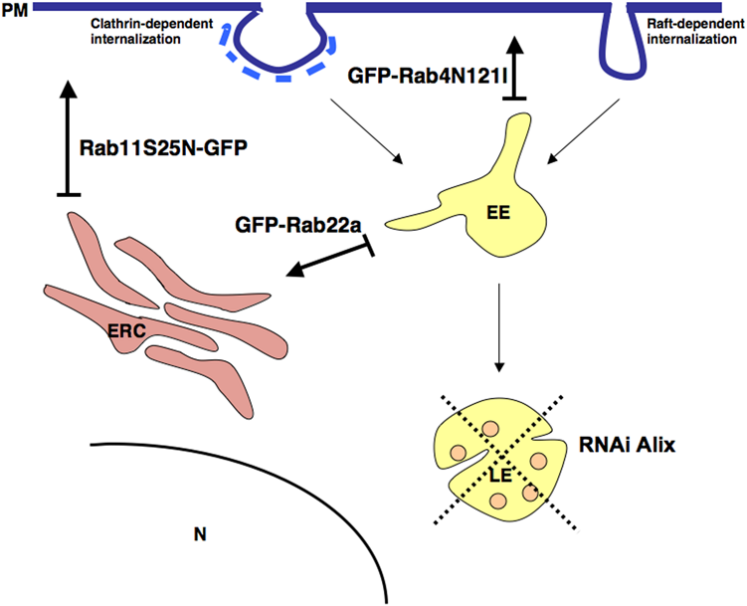
Schematic presentation of the effects of different treatments on endocytic pathways. Cargo internalized by clathrin-dependent or raft-dependent pathways is first delivered to early endosomes (EE), where sorting towards either recycling pathway (ERC) or degradative pathway (LE/lysosomes) occurs. Cargo destined to recycle can return to the plasma membrane (PM) either directly from the EE by a Rab4-controlled pathway, or it can undergo Rab22-dependent sorting to the endosomal recycling compartment (ERC). From ERC cargo is returned to PM by Rab11-dependent pathway. Cargo destined for degradation is first delivered to late endosomes (LE) and then to lysosomes, where final degradation occurs. LE act as a second sorting platform in the cell, since from there cargo can still be diverted towards recycling pathway. In order to specifically inhibit direct PM recycling from EE we expressed dominant negative Rab4 mutant coupled to GFP (GFP-Rab4N121I). Sorting to the ERC was impaired by overexpression of wild-type Rab22a (GFP-Rab22a), while export from the ERC to PM was impaired by expression of dominant-negative Rab11 coupled to GFP (Rab11S25N-GFP). To impair late endosomal pathway we affected biogenesis of LE by downregulating Alix using RNAi technology, which resulted in lower number of LE.

Because characterization of the intracellular localization of different PrP forms can provide important clues about the compartment where PrP^C^ to PrP^Sc^ conversion occurs, we reassessed the subcellular distribution of PrP^C^ and PrP^Sc^ in three different neuronal cell lines infected with different prion strains (ScGT1 infected with RML, ScCAD infected with 139A and ScN2a infected with 22L), which have been widely used as cellular models for prion infection [26],[27]. To clearly distinguish between PrP^C^ and PrP^Sc^ we employed advanced imaging technology complemented by quantitative image analysis, allowing us to define the relative amounts of PrP^C^ and PrP^Sc^ in each subcellular compartment after treating the cells with Gnd in immunofluorescence experiments (Figure S1, Methods and [28]). A similar approach, based on thresholding of the lower PrP^C^-derived fluorescence in order to extract only the higher fluorescence signal from PrP^Sc^ was recently reported by Veith and collegues in N2a cells [46]. In agreement with the results from this group and in contrast to previous reports in ScN2a and ScGT1-7 cells in which standard immunofluorescence approaches were utilized [13],[17],[32], we found only a small amount of PrP^Sc^ in LEs, arguing against the involvement of this compartment in PrP^Sc^ production. Instead, by quantitative fluorescence analysis we observed a preferential localization of PrP^Sc^ in the endosomal recycling compartment of all three cells lines tested (Figure 1 and Figure S2). In support of these findings, a similar localization was recently observed using cryo-immunogold electron microscopy on hippocampal sections from mice infected with the RML prion strain [12]. These observations prompted us to further assess the involvement of the endocytic pathways and specifically that of the endosomal recycling compartment in PrP^Sc^ conversion. To this aim we selectively inhibited PrP trafficking through the different endocytic compartments using both pharmacological and reverse genetic approaches in infected cells (see Figure 8) and analyzed PrP^Sc^ levels under the different experimental conditions. We demonstrated that EEs are not involved in PrP^Sc^ production. Indeed, when we blocked PrP exit from EEs the levels of PrP^Sc^ were drastically reduced and an accumulation of PrP^C^ in EEs was observed (Figures 2A, 2C, 5A, 5B, S8B, S8C and S8D). Besides ruling out the involvement of EEs in the conversion process, these data indicate that PrP^C^ must exit EEs in order to be converted. Furthermore, in line with previous observations [4] recycling from EEs to the cell surface does not seem to play an important role in PrP^Sc^ production (Figure S6). Therefore we analyzed the sorting from EE to LE and/or to the recycling compartment. By specifically reducing the number of LE by Alix depletion (Figure 4 and Figure S5) we demonstrated that LE are not involved in PrP^Sc^ production. In contrast, PrP sorting from EEs to the ERC seems to be the crucial event in the conversion process. In particular, we found that PrP^Sc^ levels are drastically reduced when trafficking from EEs to the ERC is specifically impaired (Figure 5 and Figure S8). We clearly demonstrate that this is not a cell-type-specific effect. Indeed, we observed a decrease in PrP^Sc^ when we overexpressed Rab22a in both ScGT1 (Figure 5) and ScCAD (Figure S8) cells, where Rab22a has a clear effect in inhibiting transport from EEs to ERC. In contrast, no reduction in PrP^Sc^ was observed when Rab22a was overexpressed in ScN2a cells, where Rab22a does not control this pathway (data not shown).

In addition to PrP^Sc^, other glycosylphosphatidylinositol (GPI)-anchored proteins (GPI-APs) have also been shown to be retained in the ERC and this retention is GPI-, sphingolipid- and cholesterol-dependent [47],[48]. Furthermore, retention of GPI-APs in the ERC can be physiologically relevant, as in the case of the Folate Receptor for which loss of retention in this compartment (by removing its GPI anchor or by depleting cholesterol) severely impairs folate uptake [49]. In the case of the prion protein, either removal of the GPI anchor, its replacement with a transmembrane domain or cellular cholesterol depletion impairs scrapie formation in cell lines [50],[51],[52],[53] even though the anchor seems to be dispensable for the conversion process itself [54],[55],[56]. In support of this hypothesis are the conclusions of a recent study by McNally and colleagues suggesting that the GPI anchor of PrP is important for the persistent infection of cells in vitro [57]. These authors found that neural stem cells derived from PrP null mice expressing only anchorless PrP cannot be persistently infected, although production of PK resistant PrP was detected in the first 96 hours after infection [57]. In agreement with these in vitro results, when a PrP molecule, fused to the Fc portion of human IgG1 heavy chain and lacking the GPI anchor, is expressed in Prnp^0/0^ mice it is not convertible and delays the onset of the disease when expressed together with wild type PrP [58]. Moreover, intracerebral inoculation of prions into Prnp^0/0^ mice expressing an anchorless form of PrP results in a reduced titre of infectivity, different anatomical localization of amyloid plaques and no obvious clinical signs of disease compared to wild type mice [59]. This suggests that the GPI anchor, although not essential for the conversion process, plays an important role in its efficiency in vivo. Thus, it is possible that the GPI anchor is necessary for mediating the targeting of PrP to the correct intracellular site (and/or to a propitious membrane domain) in order to sustain the conversion process. In this scenario PrP retention in the ERC might be necessary in order to concentrate prion proteins in a cholesterol enriched membrane domain for a sufficient amount of time to promote conversion. Indeed, by inhibiting PrP exit from the ERC using the Rab11 dominant-negative mutant, we found an increase in PrP^Sc^ levels, supporting the hypothesis that prion accumulation in the ERC stimulates PrP^Sc^ production. Furthermore, the observation that cholesterol redistribution from other cellular compartments to LEs, induced by U18666A treatment, impairs PrP trafficking from EEs to the ERC (Figure 2C) suggests that cholesterol is not only important for GPI-AP retention within the ERC, but might also be required for its sorting from EEs to the ERC. Given that protein sorting from EEs to the ERC is Rab22a-dependent [41],[42] it is possible that, similarly to Rab4 [60], cholesterol levels also influence Rab22a function. However, this attractive possibility remains to be explored.

The mechanism of PrP^C^ to PrP^Sc^ conversion remains unknown. It has been proposed that the conversion process requires interaction between PrP^C^ and PrP^Sc^ and is assisted by other protein(s) [1]. However, despite years of effort this protein has not been identified, partially due to the secondary responses of cells to prion infection that render analysis more difficult [61],[62]. Our results suggest that if such a protein exists it encounters PrP in the ERC, therefore narrowing down potential candidates. Alternatively, the PrP^C^ to PrP^Sc^ conversion may not be mediated by a specific protein but instead may depend on the local lipid environment.

The observation that cholesterol depletion reduces PrP^Sc^ levels has highlighted the importance of the lipid environment for scrapie production [50]. Cholesterol and glycosphingolipids are enriched in membrane microdomains, known as detergent-resistant domains (DRMs) or lipid rafts [63]. They have been implicated in various processes such as signal transduction, endocytosis and cholesterol trafficking [64]. It has been proposed that PrP^C^ to PrP^Sc^ conversion occurs within lipid rafts, since both proteins were found to reside in such domains [65]. Recent evidence suggests that lipid rafts are heterogeneous both in terms of their protein and lipid content, and can be localized to different regions of the cell [64]. Furthermore, it has been shown that the endocytic route of GPI-APs is cell-type dependent and correlates with their residence time in DRMs. In CHO and BHK cells, transport of GPI-APs to the ERC or to late endosomes, respectively, is accompanied by differential association to DRMs [66]. Interestingly, PrP and Thy-1, another GPI-AP, were found to reside in distinct lipid domains in rat and mouse brain [67]. Moreover, it has been shown that Thy-1 associates with rafts only transiently [68] and that raft partitioning, or raft residence time, can be modulated by subtle changes in lipid composition [69]. Therefore, a cholesterol-dependent retention mechanism in the ERC could facilitate the efficient conversion of native prion protein into the scrapie form, by segregating it into distinct membrane domains or aggregates. Interestingly, ERC membranes have been shown to be enriched in cholesterol in different cells [24],[70],[71]. Furthermore, PrP association with specific lipid domains in different cellular contexts may influence its cellular transport and thereby determine the differential cellular susceptibility to prion infection. In conclusion, our data, based on observations in three different cell models of prion infection, indicate that the ERC is **a** likely candidate for the intracellular site of prion conversion. Furthermore, the fact that these results were consistent for two different prion strains (RML and 22L) suggests that this is a general mechanism. Although we cannot completely exclude contributions by other compartments to prion conversion, recent evidence, showing a localization of PrP^Sc^ in the recycling endosomes of primary hippocampal neurons derived from infected brains [12] supports our hypothesis, strengthening the case for the role of the ERC in prion conversion in infected mouse models. These results open the door to more targeted approaches to study the factors involved in this central event of the disease and to develop better therapeutic strategies.

## Methods

### Cell culture, reagents, antibodies, plasmids and oligos

GT1-1 cells (gift of Dr. Mellon P., University of California, San Diego, USA) were infected with RML prion strain (gift of Dr. Korth K., Heinrich Heine University Düsseldorf, Germany) and ScN2a cells infected with 22L prion strain (gift of Dr. Korth K., Heinrich Heine University Düsseldorf, Germany) were cultured in DMEM with addition of 10% FCS (Invitrogen). ScCAD cells infected with 139A prion strain (gift of Dr. Laude H., Institut National de la Recherche Agronomique, Jouy-en-Josas, France) were cultured in DMEM:F12 (Invitrogen) with addition of 10% FBS. U18666A was purchased from Calbiochem. All other chemicals were purchased from Sigma. SAF32 and SAF61 monoclonal antibodies were purchased from SPI-BIO, while POM1 monoclonal antibodies were purchased from Dr. A. Aguzzi (Institute of Neuropathology, University Hospital Zurich, Zurich, Switzerland). Anti-tubulin monoclonal antibodies were from Sigma. Anti-flotillin-1 monoclonal antibodies were from BD Transduction Laboratories. Anti-GFP antibodies and all the fluorescently labeled secondary antibodies were purchased from Invitrogen (Molecular Probes), as well as Lysosensor, fluorescently tagged transferrin and dextran. Anti-EEA-1 antibodies were kind gift of Dr. M. Zerial (Max Planck Institute of Molecular Cell Biology and Genetics, Dresden, Germany) and anti-LBPA antibodies were gift from Dr. J. Gruenberg (University of Geneva, Geneva, Switzerland). Anti-Alix antibodies were gift from Dr. R. Sadoul (Neurodégénérescence et Plasticité, E0108, INSERM/Université Joseph Fourier, Grenoble, France). Anti-Rab22a antibodies and GFP-Rab22a construct were kind gift of Dr. L. Mayorga (Laboratorio de Biología Celular y Molecular, Instituto de Histología y Embriología (IHEM-CONICET), Facultad de Ciencias Médicas, Universidad Nacional de Cuyo, Mendoza, Argentina). Rab11wt-GFP and Rab11S25N-GFP were gift of Dr. S.Mayor (National Centre for Biological Science (TIFR), Bellary Road, Bangalore 560 065, India). Alix siRNA oligos were published previously [39] and purchased from Dharmacon. All the other siRNA used in the study were predesigned ON TARGETplus SMARTpool from Dharmacon.

### Treatment of ScGT1 cells with U18666A

U18666A was reconstituted according to producer's instructions and used in DMEM+10% FCS at 5 µM concentration for ScGT1-1 cells or 1 µM concentration for ScCAD and ScN2a cells. During the 6 day-treatment, medium containing U18666A was changed every 2 days. To analyze PrP levels on western blot cells were lysed and lysates were either treated or not with Proteinase K as described. PrP was revealed by SAF61 antibodies. For immunofluorescence analysis cells were washed after the 6 day treatment, and processed as described below in Immunofluorescence analysis.

### Transient transfections

ScGT1-1 cells were transfected at 50% confluence using FuGENE6 (Roche Diagnostic) for DNA constructs according to manufacturers protocol. Transfection of siRNA in ScGT1-1 was done using HiPerFect (Qiagen). To downregulate Alix and Rab22a, 250 nM oligo and 500 nM oligo respectively was used with 10 µl of HiPerFect per 60 mm dish, while 3 µl of HiPerFect was used per well for cells grown in 24-well plate. Hyperfect was mixed with siRNA in DMEM without FBS, incubated for 10 min at room temperature and added to the cells. Transfection of ScCAD and ScN2a cells with both DNA constructs and siRNA was done using Lipofectamine 2000 (Invitrogen), according to producer's protocol. In order to detect the effect on PrP^Sc^ levels, both silencing and overexpression of proteins were required during 6-day period. Therefore in all the experiments siRNA and plasmids, except of pEGFP were transfected twice (3 days post-transfection a second transfection was performed) during 6 days.

### Endocytosis of fluorescently labelled transferrin and dextran

ScGT1-1, ScCAD and ScN2a cells (200000) were grown on coverslips for 2 days. For steady state localization, 50 µg/ml of Alexa-labeled transferrin was added to the medium for 15 minutes at 37°C. In pulse and chase experiment 50 µg/ml of transferrin was added to the medium for 15 min at 37°C. The cells were extensively washed with PBS and Alexa-labeled transferrin was chased out of the cells with the excess (5 mg/ml) of unlabeled transferrin for indicated time period.

To follow dextran endocytosis by immunofluorescence, cells were first incubated in 10% FBS at 4°C for 30 min followed by 30 min incubation with 3 mg/ml of Alexa-dextran at 37°C. Cells were then extensively washed with PBS and chased in the medium deprived from dextran for additional 3 h at 37°C.

### Immunofluorescence analysis

For immunofluorescence analysis, cells were fixed with 2% paraformaldehyde for 30 min unless differently indicated and permeabilized with 0,1% of Triton X-100/PBS. To analyze PrP^Sc^ cells were additionally incubated in 6 M guanidine-hydrochloride for 10 min after permeabilization. Cells were then blocked in 2% BSA for 30 min unless differently indicated, following by 30 min-incubation with primary and secondary antibodies respectively. For immunofluorescence analysis of Alexa-labeled dextran cells were fixed in 4% PFA for 4 h and further processed as described. When filipin staining was used, cells were fixed with 4% PFA for 60 min and blocked with 0,2% BSA/PBS. Filipin (250 µg/ml) was added to blocking solution and additional 30 min-incubation was performed after incubation with secondary antibodies. Immunofluorescence was analyzed by high-resolution wide-field microscope Marianas (Intelligent Imaging Innovations) using a 63× oil objective.

### Image acquisition, processing and quantification

When PrP^Sc^ was analyzed, the auto-scaling (min/max) of signal detection was used to record only maximal signal intensities. Briefly, the exposure time (100 ms) used to detect PrP^Sc^ fluorescence was insufficient to detect significant signal coming from PrP^C^. The camera settings were then adjusted to record only the range of PrP^Sc^-derived fluorescence signal. The images were deconvolved using constrained iterative algorithm in Slidebook software (Intelligent Imaging Innovations). Colocalization was quantified by intensity correlation coefficient-based (ICCB) analysis using Imaris software (Bitplane) or JACoP (http://rsb.info.nih.gov/ij/plugins/track/jacop.htlm). Statistical analysis of the correlation of the intensity values of either green and red pixels or blue and red pixels in dual-channel image was performed using Pearson's and Menders's coeficient and Van Steensel's approach [72]. The amount of total fluorescent signal in one channel overlapping with the total fluorescent signal in the other channel was presented. To quantify the number of LBPA and Lysosoensor positive vesicles in control cells or cells transfected with Alix siRNA, the vesicles were counted by particle analysis using Image J software (http://rsb.info.nih.gov/ij).

### Detergent Resistant Microdomain (DRM) analysis by sucrose density gradients

Control ScGT1 and U18666A-treated cells grown to confluence in 150 mm dishes were harvested in cold PBS and resuspended in 1 ml lyses buffer (1% TX-100, 10 mM Tris-HCl pH 7.5, 150 mM NaCl, 5 mM EDTA), left on ice for 20 minutes and passed 10 times through 22-gauge needles. Lysates were mixed with an equal volume of 85% sucrose (w/v) in 10 mM Tris-HCl pH 7.5, 150 mM NaCl, 5 mM EDTA, placed at the bottom of a discontinuous sucrose gradient (30–5%) in the same buffer and ultracentrifuged at 200,000 g for 17 hours at 4°C in an ultracentrifuge (SW41 rotor from Beckman Instruments, Fullerton, CA, USA). Twelve fractions were harvested from the top of the gradient. A white light-scattering band identified in fraction 5 at the interface between 5 and 30% sucrose, contained DRM domains. Samples were TCA precipitated and proteins were analyzed by western blotting.

### Protein analysis

Cells were grown to confluence in 60 mm dishes and lysed in 500 µl of Lyses buffer (0,5% triton X-100, 0,5% DOC, 100 mM NaCl, 10 mM Tris-HCl pH 8). To analyze PrP^Sc^ by western blot, lysates (500 µg of protein) were treated with 20 µg/ml of Proteinase K (PK) for 30 min on 37°C and protein content was pelleted by centrifugation at 14000 rpm and 4°C for 1 hr. Pellets were resuspended in Laemmli buffer and proteins were analyzed by western blotting. In contrast to PrP^C^, which is completely degraded by PK, only partial degradation of PrP^Sc^ occurs in this condition [73]. Other proteins, including total PrP were analyzed by western blotting from 25 µg of total lysate.

### Statistical analysis

T-test was used for statistical analysis of the data. The differences were considered significant when p<0.05.

## Supporting Information

Figure S1 Acquisition adjustments to detect only PrP^Sc^ by immunofluorescence. (A) Control ScGT1 cells and cells treated with 1 µg/ml of dextran sulphate for 6 days were lysed, incubated or not with 20 µg/ml of Proteinase K (PK) and levels of total PrP or PK resistant PrP (PrP^Sc^) were analyzed on western blot using SAF61 mAb. Note that no PrP^Sc^ could be observed upon treatment with dextran sulphate. (B) Control ScGT1 cells and (C) cells treated with 1 µg/ml of dextran sulphate for 6 days were fixed, permeabilized and treated with guanidine-hydrochloride as described in Methods. PrP^Sc^ was revealed by SAF32 mAb and analyzed by high resolution wide-field microscope Marianas (Intelligent Imaging Innovations). Exposure times used to acquire images were 100 ms for PrP^Sc^ and 500 ms for PrP^C^. Auto scaling option (min/max) was used to permit detection of only maximal signal intensities. Note that those settings permitted to detect only PrP^Sc^ signal with 100 ms as seen in control cells, which was completely absent in cells cured by dextran sulphate treatment (compare b and c upper panels).
(0.96 MB TIF)

Figure S2 Steady-state distribution of PrP^Sc^ in ScCAD and ScN2a cells infected with different prion strains. PrP^Sc^ was revealed using SAF32 mAb, after denaturation with Gnd as described in Methods and colocalization with Giantin (Golgi), Lamp-1 (lysosomes), EEA-1 (early endosomes) and Alexa 488-transferrin (Tfn); marker for the perinuclear recycling compartment) was analyzed. Yellow colour indicates colocalization. Note that PrP^Sc^ significantly colocalizes with Tfn in the ERC of both cell lines. Scale bars 10 µm.
(1.57 MB TIF)

Figure S3 U18666A treatment reduces PrP^Sc^ levels in ScN2a cells and impairs trafficking of Tfn and PrP, without affecting PrP^C^ distribution in detergent resistant domains (DRMs). (A) Control ScN2a cells and cells treated with 1 µM U18666A for 6 days were lysed and levels of total PrP or PrP^Sc^ were analyzed on western blot using SAF61 mAb. To reveal PrP^Sc^ lysates were incubated with 20 µg/ml of Proteinase K (PK). Note that no PrP^Sc^ could be observed in U18666A treated cells. (B) Lysates from control and U18666A treated ScN2a cells were applied on sucrose gradient and ultracentrifuged at 200000 g for 16 hr. Twelve fractions were collected and proteins were precipitated using 10% of trichloroacetic acid. PrP and flotillin-1 contents in each fraction were analyzed on western blot using SAF61 mAb and flotillin-1 mAb. Fractions 4–7 correspond to detergent resistant membranes (DRM) based on distribution of flotillin-1, which is mainly present in DRMs. In ScN2a cells PrP is distributed in DRMs and U18666A treatment does not change its distribution. (C) Steady-state localization of PrP^C^ was analyzed in control (ctrl) and U18666A-treated cells after 6 days of treatment. The effect of the treatment was assessed by cholesterol accumulation in LE based on filipin staining shown in blue. Yellow colour represents colocalization between PrP^C^ and EEA-1. Note that in control condition PrP^C^ and cholesterol were exclusively localized at the cell surface, while upon U18666A treatment, cholesterol was redistributed to LE and PrP^C^ was enriched in EEA-1 positive EE. (D) Alexa 488-Tfn was internalized for 15 min and its subcellular distribution was analyzed in control and U18666A-treated cells. Yellow colour represents colocalization between EEA-1 and Alexa 488-Tfn. Note that in U18666A-treated cells Alexa 488-Tfn is confined to EE and does not accumulate in the ERC. Scale bars 10 µm.
(2.26 MB TIF)

Figure S4 U18666A blocks EE to LE traffic in ScGT1 cells. (A) To study traffic from early (EE) to late endosomes (LE), control ScGT1 cells were either incubated with Alexa 488-dextran for 15 min and then fixed, permeabilized and immunolabeled for EEA-1, or additionally incubated in a dextran-free medium for 3 h and then fixed, permeabilized and immunolabeled for LBPA. Yellow colour and arrowheads indicate colocalization. In control cells a diffuse cholesterol distribution was revealed by fillipin staining. Inset represents magnification of the boxed area. Upon 15 min pulse dextran was internalized into EE and then was chased to LE upon subsequent incubation in dextran-free medium. (B) The experiment described in (A) was performed in ScGT1 cells treated with 5 µM U18666A. Characteristic cholesterol laden late endosomes were revealed with both filipin staining (blue) and immunolabeling for LBPA (red, lower panels). Magenta colour represents colocalization between filipin and LBPA in lower panels. Yellow colour and arrowheads indicate colocalization between dextran and EEA-1. Treated cells were able to internalize dextran into EE upon 15 min pulse, but in contrast to control cells they were not able to deliver dextran to the LE after 3 hr chase period. Scale bars 10 µm.
(2.47 MB TIF)

Figure S5 Alix downregulation in ScGT1 and ScCAD cells causes reduction in the number of late endosomes/lysosomes without affecting PrP^Sc^ levels. ScGT1 and ScCAD cells were transfected with either control oligo (ctrl ol) or RNAi against Alix (siAlix) during six days. (A) ScGT1 cells were let to internalize Lysosensor, which emits fluorescence only in acidic compartments followed by fixation, permeabilization and immunolabeling for LBPA. Scale bars 10 µm. The number of Lysosensor and LBPA positive vesicles was analyzed by Image J software. Results are presented as number of vesicles (mean±s.e.m) counted in 60 cells from 2 different experiments. Significant decrease in number of Lysosensor and LBPA positive vesicles was observed in Alix depleted ScGT1 cells when compared to control cells (p = 0,0011, t-test). (B) Alix and PrP^Sc^ levels were analysed by western blot in lysates from ScCAD cells transfected with Alix siRNA for 6 days. Levels of PrP^Sc^ were analyzed after the treatment with 20 µg/ml of PK using SAF61 mAb. PrP^Sc^ levels were not affected upon Alix downregulation. Similar results are obtained for ScGT1 cells (Figure 4).
(1.26 MB TIF)

Figure S6 Expression of GFP-Rab4wt or GFP-Rab4N121I in ScGT1 and ScN2a cells does not influence PrP^Sc^ levels. Control ScGT1 and ScN2a cells and cells transfected with GFP-Rab4 constructs for 6 days were lysed and levels of GFP-Rab4 were analyzed on western blot using anti-GFP Abs. Bands corresponding to GFP-Rab4wt and GFP-Rab4N121I were marked by arrows. Levels of PrP^Sc^ were analyzed after the treatment with 20 µg/ml of PK using SAF61 mAb.
(0.21 MB TIF)

Figure S7 Rab22a regulates transferrin sorting to ERC and PrP^Sc^ production in ScGT1 cells. (A) Overexpression of GFP-Rab22a in ScGT1 cells inhibits Tfn sorting to ERC. ScGT1 cells were transfected with GFP-Rab22a and allowed to internalize Alexa 546-transferrin (Tfn) for 5 and 15 min. Yellow colour and arrowheads indicate colocalization. Scale bars 10 µm. The quantification results (mean±s.e.m, n = 36) are presented as % of total signal in red (Tfn) colocalizing with GFP-Rab22a (green). Increase of Tfn (p = 0,0099, t-test) in EE of GFP-Rab22a expressing cells was detected. Note that Tfn was not able to reach ERC after 15 min internalization. (B) Rab22a depletion reduces PrP^Sc^ level in ScGT1 cells. Rab22a and PrP^Sc^ levels were analyzed by western blot in lysates from untransfected cells (ctrl), or cells transfected with either control oligo (ctrl ol) or RNAi against Rab22a (siRab22) for 6 days. To reveal PrP^Sc^ the lysates were treated with 20 µg/ml of Proteinase K (PK) and SAF61 mAb was used on western blot. Around 60% downregulation in Rab22a level was observed in cells transfected with siRab22, 6 days post-transfection. The band corresponding to Rab22a is marked by an arrow. Asterisk indicates unspecific band as a control for equal loading. PrP^Sc^ levels were quantified and the results (mean±s.e.m, n = 3 experiments) are presented as % of PrP^Sc^ level in control, untransfected cells, which is considered as 100%. Around 50% reduction in PrP^Sc^ levels was observed in Rab22a depleted cells (p = 0,032, t-test).
(1.97 MB TIF)

Figure S8 Overexpression of GFP-Rab22a affects cellular distribution of Tfn and PrP^C^ and reduces PrP^Sc^ levels in ScCAD cells. (A) ScCAD cells transfected with GFP-Rab22a for 6 days were fixed and immunolabeled for EEA-1 (upper panels). In the parallel experiment (lower panels) transfected cells were let to internalize Alexa 546-Tfn for 15 min. Yellow colour represents colocalization between GFP-Rab22a and EEA-1 or GFP-Rab22a and Tfn. GFP-Rab22a was colocalizing with EEA-1 in EE, but it was also present in tubular structures (arrowheads) and EEA-1 negative vesicles (arrows). In contrast to control cells in GFP-Rab22a-expressing cells Tfn was not accumulating in the ERC, but was distributed instead in GFP-Rab22a positive compartment. (B) Cells transfected with GFP-Rab22a were fixed and immunolabeled for PrP^C^ using Saf32 mAb and EEA-1 using anti-EEA-1 Ab. White colour and arrowheads represents colocalization between PrP^C^, GFP-Rab22a and EEA-1. (C) GFP and GFP-Rab22a in transfected ScGT1 cells were analyzed on western blot using anti-GFP Abs. Levels of PrP^Sc^ (PK+) and total PrP (PK−) were analyzed on western blot. (D) PrP^Sc^ was additionally analyzed by immunofluorescence in GFP-Rab22a transfected cells upon Gnd denaturation and immunostaining with POM-1 mAb. While PrP^Sc^ was revealed in control cells, no signal for PrP^Sc^ was observed by both western blot analysis and immunofluorescence in GFP-Rab22a-expressing cells. Scale bars 10 µm.
(1.69 MB TIF)

Figure S9 Rab11wt-GFP and Rab11S25N-GFP are partially localized in the ERC and in peripheral vesicles. ScGT1 cells were transfected with Rab11 constructs for 6 days. Alexa 546-transferrin was internalized for 15 min to label ERC. Yellow colour represents colocalization. Scale bars 10 µm.
(1.69 MB TIF)

## References

[1] Prusiner SB (1998). Prions.. Proc Natl Acad Sci U S A.

[2] Campana V, Sarnataro D, Zurzolo C (2005). The highways and byways of prion protein trafficking.. Trends Cell Biol.

[3] Borchelt DR, Taraboulos A, Prusiner SB (1992). Evidence for synthesis of scrapie prion proteins in the endocytic pathway.. J Biol Chem.

[4] Beranger F, Mange A, Goud B, Lehmann S (2002). Stimulation of PrP(C) retrograde transport toward the endoplasmic reticulum increases accumulation of PrP(Sc) in prion-infected cells.. J Biol Chem.

[5] Galvan C, Camoletto PG, Dotti CG, Aguzzi A, Ledesma MD (2005). Proper axonal distribution of PrP(C) depends on cholesterol-sphingomyelin-enriched membrane domains and is developmentally regulated in hippocampal neurons.. Molecular and cellular neurosciences.

[6] Shyng SL, Huber MT, Harris DA (1993). A prion protein cycles between the cell surface and an endocytic compartment in cultured neuroblastoma cells.. J Biol Chem.

[7] Sunyach C, Jen A, Deng J, Fitzgerald KT, Frobert Y (2003). The mechanism of internalization of glycosylphosphatidylinositol-anchored prion protein.. EMBO J.

[8] Grigoriev V, Escaig-Haye F, Streichenberger N, Kopp N, Langeveld J (1999). Submicroscopic immunodetection of PrP in the brain of a patient with a new-variant of Creutzfeldt-Jakob disease.. Neuroscience letters.

[9] Fournier JG, Escaig-Haye F, Grigoriev V (2000). Ultrastructural localization of prion proteins: physiological and pathological implications.. Microscopy research and technique.

[10] Jeffrey M, Goodsir CM, Bruce ME, McBride PA, Scott JR (1994). Infection-specific prion protein (PrP) accumulates on neuronal plasmalemma in scrapie-infected mice.. Annals of the New York Academy of Sciences.

[11] Barmada SJ, Harris DA (2005). Visualization of prion infection in transgenic mice expressing green fluorescent protein-tagged prion protein.. J Neurosci.

[12] Godsave SF, Wille H, Kujala P, Latawiec D, DeArmond SJ (2008). Cryo-immunogold electron microscopy for prions: toward identification of a conversion site.. The Journal of neuroscience : the official journal of the Society for Neuroscience.

[13] Pimpinelli F, Lehmann S, Maridonneau-Parini I (2005). The scrapie prion protein is present in flotillin-1-positive vesicles in central- but not peripheral-derived neuronal cell lines.. Eur J Neurosci.

[14] Magalhaes AC, Silva JA, Lee KS, Martins VR, Prado VF (2002). Endocytic intermediates involved with the intracellular trafficking of a fluorescent cellular prion protein.. J Biol Chem.

[15] Taraboulos A, Serban D, Prusiner SB (1990). Scrapie prion proteins accumulate in the cytoplasm of persistently infected cultured cells.. J Cell Biol.

[16] Caughey B, Raymond GJ, Ernst D, Race RE (1991). N-terminal truncation of the scrapie-associated form of PrP by lysosomal protease(s): implications regarding the site of conversion of PrP to the protease-resistant state.. J Virol.

[17] McKinley MP, Taraboulos A, Kenaga L, Serban D, Stieber A (1991). Ultrastructural localization of scrapie prion proteins in cytoplasmic vesicles of infected cultured cells.. Lab Invest.

[18] Vey M, Pilkuhn S, Wille H, Nixon R, DeArmond SJ (1996). Subcellular colocalization of the cellular and scrapie prion proteins in caveolae-like membranous domains.. Proc Natl Acad Sci U S A.

[19] Caughey B, Raymond GJ (1991). The scrapie-associated form of PrP is made from a cell surface precursor that is both protease- and phospholipase-sensitive.. The Journal of biological chemistry.

[20] Taraboulos A, Raeber AJ, Borchelt DR, Serban D, Prusiner SB (1992). Synthesis and trafficking of prion proteins in cultured cells.. Molecular biology of the cell.

[21] Gilch S, Winklhofer KF, Groschup MH, Nunziante M, Lucassen R (2001). Intracellular re-routing of prion protein prevents propagation of PrP(Sc) and delays onset of prion disease.. EMBO J.

[22] Mayor S, Pagano RE (2007). Pathways of clathrin-independent endocytosis.. Nature reviews Molecular cell biology.

[23] Gruenberg J, Maxfield FR (1995). Membrane transport in the endocytic pathway.. Current opinion in cell biology.

[24] Maxfield FR, McGraw TE (2004). Endocytic recycling.. Nature reviews Molecular cell biology.

[25] Zerial M, McBride H (2001). Rab proteins as membrane organizers.. Nature reviews Molecular cell biology.

[26] Mahal SP, Baker CA, Demczyk CA, Smith EW, Julius C (2007). Prion strain discrimination in cell culture: the cell panel assay.. Proceedings of the National Academy of Sciences of the United States of America.

[27] Vilette D (2008). Cell models of prion infection.. Veterinary research.

[28] Gousset K, Schiff E, Langevin C, Marijanovic Z, Caputo A (2009). Prions hijack tunnelling nanotubes for intercellular spread.. Nat Cell Biol.

[29] Pan T, Wong P, Chang B, Li C, Li R (2005). Biochemical fingerprints of prion infection: accumulations of aberrant full-length and N-terminally truncated PrP species are common features in mouse prion disease.. J Virol.

[30] Silveira JR, Raymond GJ, Hughson AG, Race RE, Sim VL (2005). The most infectious prion protein particles.. Nature.

[31] Veith NM, Plattner H, Stuermer CA, Schulz-Schaeffer WJ, Burkle A (2009). Immunolocalisation of PrPSc in scrapie-infected N2a mouse neuroblastoma cells by light and electron microscopy.. Eur J Cell Biol.

[32] Arnold JE, Tipler C, Laszlo L, Hope J, Landon M (1995). The abnormal isoform of the prion protein accumulates in late-endosome-like organelles in scrapie-infected mouse brain.. J Pathol.

[33] Baron GS, Wehrly K, Dorward DW, Chesebro B, Caughey B (2002). Conversion of raft associated prion protein to the protease-resistant state requires insertion of PrP-res (PrP(Sc)) into contiguous membranes.. EMBO J.

[34] Sobo K, Le Blanc I, Luyet P-P, Fivaz M, Ferguson C (2007). Late endosomal cholesterol accumulation leads to impaired intra-endosomal trafficking.. PLoS ONE.

[35] Mayran N, Parton RG, Gruenberg J (2003). Annexin II regulates multivesicular endosome biogenesis in the degradation pathway of animal cells.. EMBO J.

[36] Kobayashi T, Beuchat MH, Lindsay M, Frias S, Palmiter RD (1999). Late endosomal membranes rich in lysobisphosphatidic acid regulate cholesterol transport.. Nat Cell Biol.

[37] Klingenstein R, Lober S, Kujala P, Godsave S, Leliveld SR (2006). Tricyclic antidepressants, quinacrine and a novel, synthetic chimera thereof clear prions by destabilizing detergent-resistant membrane compartments.. J Neurochem.

[38] te Vruchte D, Lloyd-Evans E, Veldman RJ, Neville DCA, Dwek RA (2004). Accumulation of glycosphingolipids in Niemann-Pick C disease disrupts endosomal transport.. The Journal of biological chemistry.

[39] Matsuo H, Chevallier J, Mayran N, Le Blanc I, Ferguson C (2004). Role of LBPA and Alix in multivesicular liposome formation and endosome organization.. Science.

[40] Roberts M, Barry S, Woods A, van der Sluijs P, Norman J (2001). PDGF-regulated rab4-dependent recycling of alphavbeta3 integrin from early endosomes is necessary for cell adhesion and spreading.. Curr Biol.

[41] Magadan JG, Barbieri MA, Mesa R, Stahl PD, Mayorga LS (2006). Rab22a regulates the sorting of transferrin to recycling endosomes.. Mol Cell Biol.

[42] Weigert R, Yeung AC, Li J, Donaldson JG (2004). Rab22a regulates the recycling of membrane proteins internalized independently of clathrin.. Molecular biology of the cell.

[43] Seglen PO (1983). Inhibitors of lysosomal function.. Methods Enzymol.

[44] Ullrich O, Reinsch S, Urbe S, Zerial M, Parton RG (1996). Rab11 regulates recycling through the pericentriolar recycling endosome.. J Cell Biol.

[45] Urbe S, Huber LA, Zerial M, Tooze SA, Parton RG (1993). Rab11, a small GTPase associated with both constitutive and regulated secretory pathways in PC12 cells.. FEBS Lett.

[46] Veith NM, Plattner H, Stuermer CAO, Schulz-Schaeffer WJ, Burkle A (2009). Immunolocalisation of PrPSc in scrapie-infected N2a mouse neuroblastoma cells by light and electron microscopy.. European journal of cell biology.

[47] Mayor S, Sabharanjak S, Maxfield FR (1998). Cholesterol-dependent retention of GPI-anchored proteins in endosomes.. EMBO J.

[48] Chatterjee S, Smith ER, Hanada K, Stevens VL, Mayor S (2001). GPI anchoring leads to sphingolipid-dependent retention of endocytosed proteins in the recycling endosomal compartment.. EMBO J.

[49] Ritter TE, Fajardo O, Matsue H, Anderson RG, Lacey SW (1995). Folate receptors targeted to clathrin-coated pits cannot regulate vitamin uptake.. Proceedings of the National Academy of Sciences of the United States of America.

[50] Taraboulos A, Scott M, Semenov A, Avrahami D, Laszlo L (1995). Cholesterol depletion and modification of COOH-terminal targeting sequence of the prion protein inhibit formation of the scrapie isoform.. J Cell Biol.

[51] Rogers M, Yehiely F, Scott M, Prusiner SB (1993). Conversion of truncated and elongated prion proteins into the scrapie isoform in cultured cells.. Proc Natl Acad Sci U S A.

[52] Caughey B, Raymond GJ (1991). The scrapie-associated form of PrP is made from a cell surface precursor that is both protease- and phospholipase-sensitive.. J Biol Chem.

[53] Priola SA, Lawson VA (2001). Glycosylation influences cross-species formation of protease-resistant prion protein.. EMBO J.

[54] Kocisko DA, Come JH, Priola SA, Chesebro B, Raymond GJ (1994). Cell-free formation of protease-resistant prion protein.. Nature.

[55] Lawson VA, Priola SA, Wehrly K, Chesebro B (2001). N-terminal truncation of prion protein affects both formation and conformation of abnormal protease-resistant prion protein generated in vitro.. J Biol Chem.

[56] Baron GS, Caughey B (2003). Effect of glycosylphosphatidylinositol anchor-dependent and -independent prion protein association with model raft membranes on conversion to the protease-resistant isoform.. J Biol Chem.

[57] McNally KL, Ward AE, Priola SA (2009). Cells Expressing Anchorless Prion Protein are Resistant to Scrapie infection.. J Virol.

[58] Meier P, Genoud N, Prinz M, Maissen M, Rulicke T (2003). Soluble dimeric prion protein binds PrP(Sc) in vivo and antagonizes prion disease.. Cell.

[59] Chesebro B, Trifilo M, Race R, Meade-White K, Teng C (2005). Anchorless prion protein results in infectious amyloid disease without clinical scrapie.. Science.

[60] Choudhury A, Sharma DK, Marks DL, Pagano RE (2004). Elevated endosomal cholesterol levels in Niemann-Pick cells inhibit rab4 and perturb membrane recycling.. Molecular biology of the cell.

[61] Fasano C, Campana V, Griffiths B, Kelly G, Schiavo G (2008). Gene expression profile of quinacrine-cured prion-infected mouse neuronal cells.. Journal of neurochemistry.

[62] Julius C, Hutter G, Wagner U, Seeger H, Kana V (2008). Transcriptional stability of cultured cells upon prion infection.. Journal of molecular biology.

[63] Simons K, Ikonen E (1997). Functional rafts in cell membranes.. Nature.

[64] Pike LJ (2004). Lipid rafts: heterogeneity on the high seas.. The Biochemical journal.

[65] Naslavsky N, Shmeeda H, Friedlander G, Yanai A, Futerman AH (1999). Sphingolipid depletion increases formation of the scrapie prion protein in neuroblastoma cells infected with prions.. J Biol Chem.

[66] Fivaz M, Vilbois F, Thurnheer S, Pasquali C, Abrami L (2002). Differential sorting and fate of endocytosed GPI-anchored proteins.. The EMBO journal.

[67] Madore N, Smith KL, Graham CH, Jen A, Brady K (1999). Functionally different GPI proteins are organized in different domains on the neuronal surface.. The EMBO journal.

[68] Sheets ED, Lee GM, Simson R, Jacobson K (1997). Transient confinement of a glycosylphosphatidylinositol-anchored protein in the plasma membrane.. Biochemistry.

[69] Dietrich C, Volovyk ZN, Levi M, Thompson NL, Jacobson K (2001). Partitioning of Thy-1, GM1, and cross-linked phospholipid analogs into lipid rafts reconstituted in supported model membrane monolayers.. Proceedings of the National Academy of Sciences of the United States of America.

[70] Hao M, Lin SX, Karylowski OJ, Wustner D, McGraw TE (2002). Vesicular and non-vesicular sterol transport in living cells. The endocytic recycling compartment is a major sterol storage organelle.. The Journal of biological chemistry.

[71] Wustner D, Herrmann A, Hao M, Maxfield FR (2002). Rapid nonvesicular transport of sterol between the plasma membrane domains of polarized hepatic cells.. The Journal of biological chemistry.

[72] van Steensel B, van Binnendijk EP, Hornsby CD, van der Voort HT, Krozowski ZS (1996). Partial colocalization of glucocorticoid and mineralocorticoid receptors in discrete compartments in nuclei of rat hippocampus neurons.. Journal of cell science.

[73] Prusiner SB, Groth DF, Bolton DC, Kent SB, Hood LE (1984). Purification and structural studies of a major scrapie prion protein.. Cell.

