# Supplementary figures and images for: Identification of an Intracellular Site of Prion Conversion
Source: PLoS Pathog. 2009 May 8;5(5):e1000426. doi: 10.1371/journal.ppat.1000426 (PMC2673690; doi:10.1371/journal.ppat.1000426)

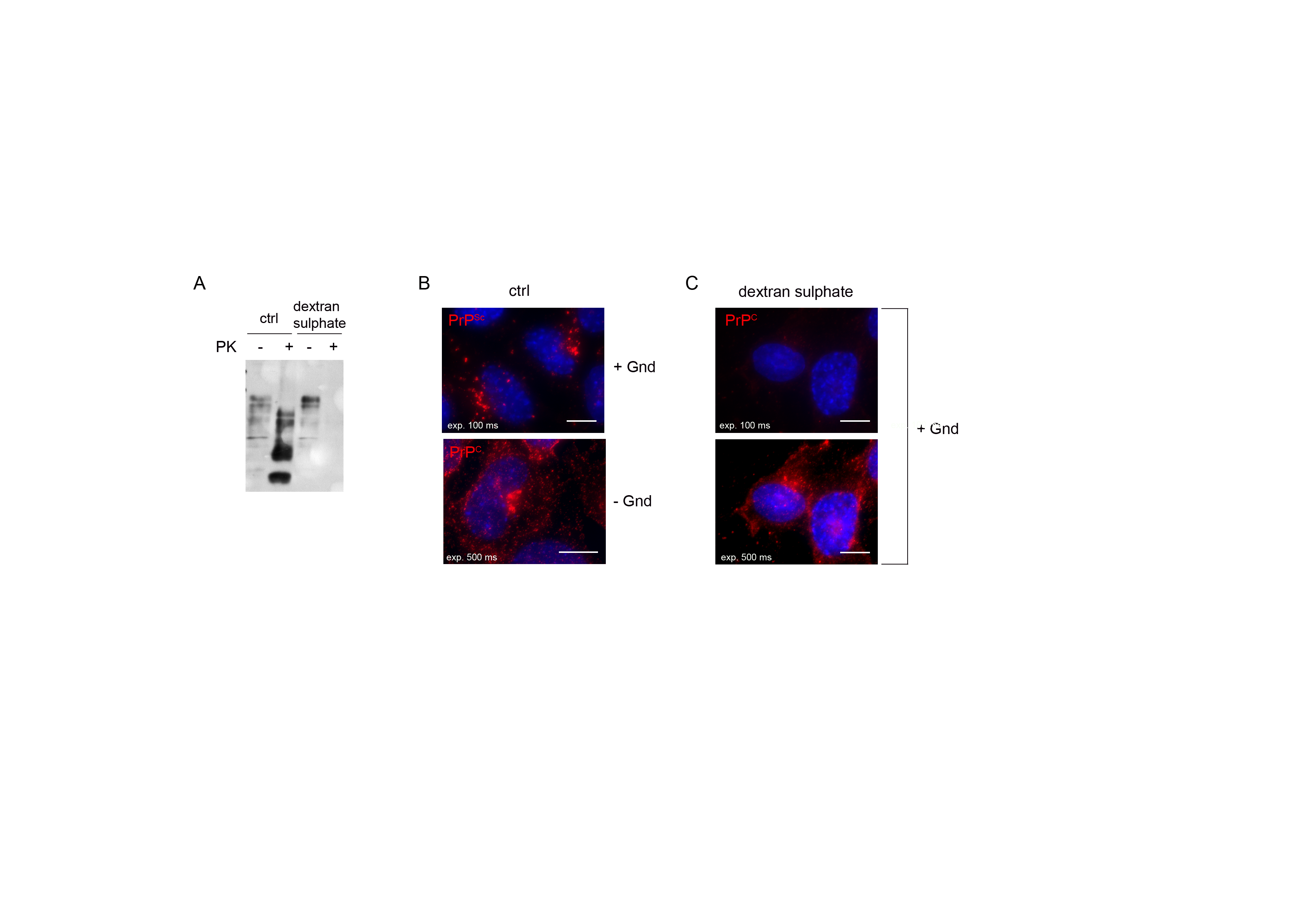

Supplement: Figure S1 — Acquisition adjustments to detect only PrPSc by immunofluorescence. (A) Control ScGT1 cells and cells treated with 1 µg/ml of dextran sulphate for 6 days were lysed, incubated or not with 20 µg/ml of Proteinase K (PK) and levels of total PrP or PK resistant PrP (PrPSc) were analyzed on western blot using SAF61 mAb. Note that no PrPSc could be observed upon treatment with dextran sulphate. (B) Control ScGT1 cells and (C) cells treated with 1 µg/ml of dextran sulphate for 6 days were fixed, permeabilized and treated with guanidine-hydrochloride as described in Methods. PrPSc was revealed by SAF32 mAb and analyzed by high resolution wide-field microscope Marianas (Intelligent Imaging Innovations). Exposure times used to acquire images were 100 ms for PrPSc and 500 ms for PrPC. Auto scaling option (min/max) was used to permit detection of only maximal signal intensities. Note that those settings permitted to detect only PrPSc signal with 100 ms as seen in control cells, which was completely absent in cells cured by dextran sulphate treatment (compare b and c upper panels). (0.96 MB TIF) [file ppat.1000426.s001.tif]

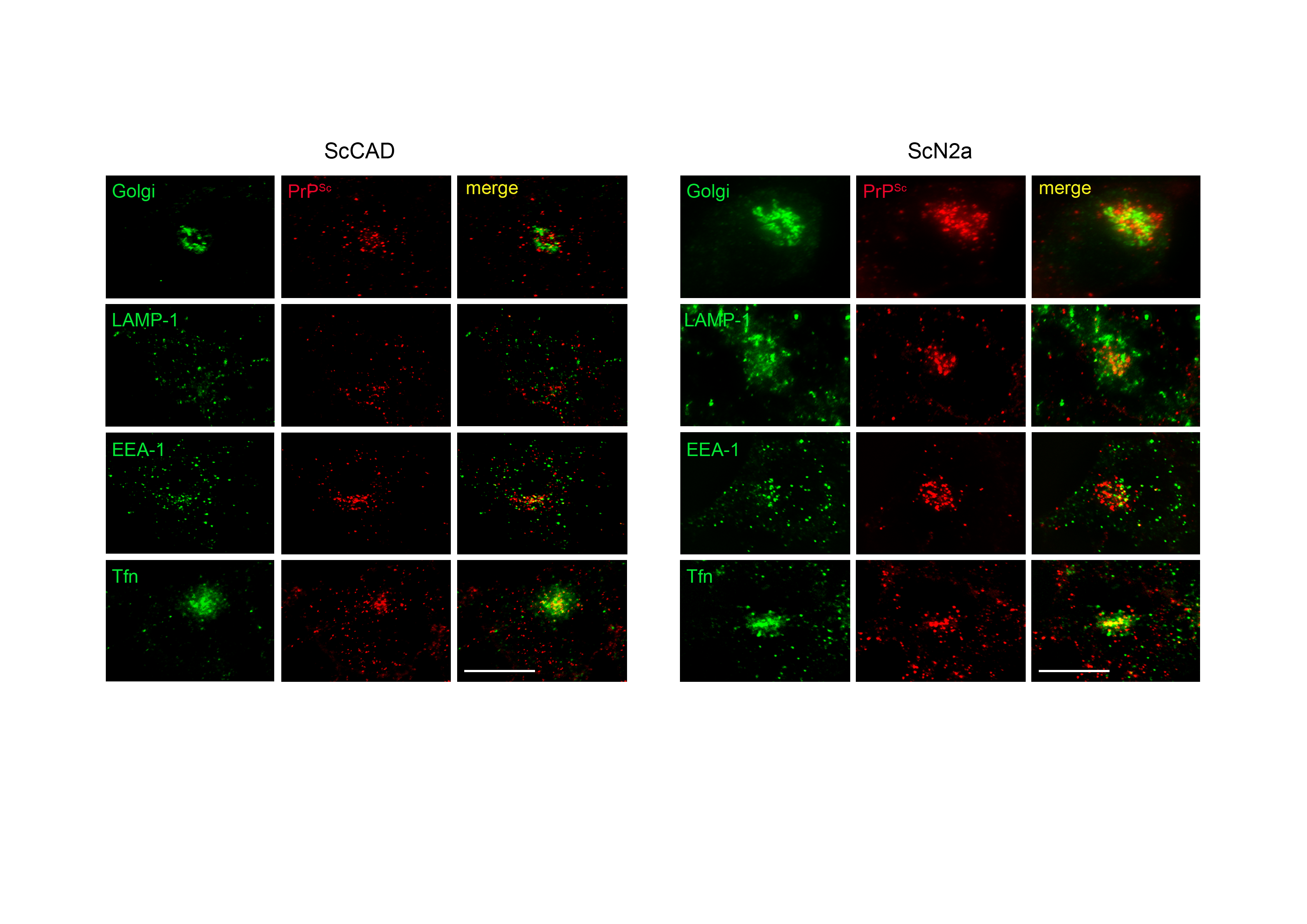

Supplement: Figure S2 — Steady-state distribution of PrPSc in ScCAD and ScN2a cells infected with different prion strains. PrPSc was revealed using SAF32 mAb, after denaturation with Gnd as described in Methods and colocalization with Giantin (Golgi), Lamp-1 (lysosomes), EEA-1 (early endosomes) and Alexa 488-transferrin (Tfn); marker for the perinuclear recycling compartment) was analyzed. Yellow colour indicates colocalization. Note that PrPSc significantly colocalizes with Tfn in the ERC of both cell lines. Scale bars 10 µm. (1.57 MB TIF) [file ppat.1000426.s002.tif]

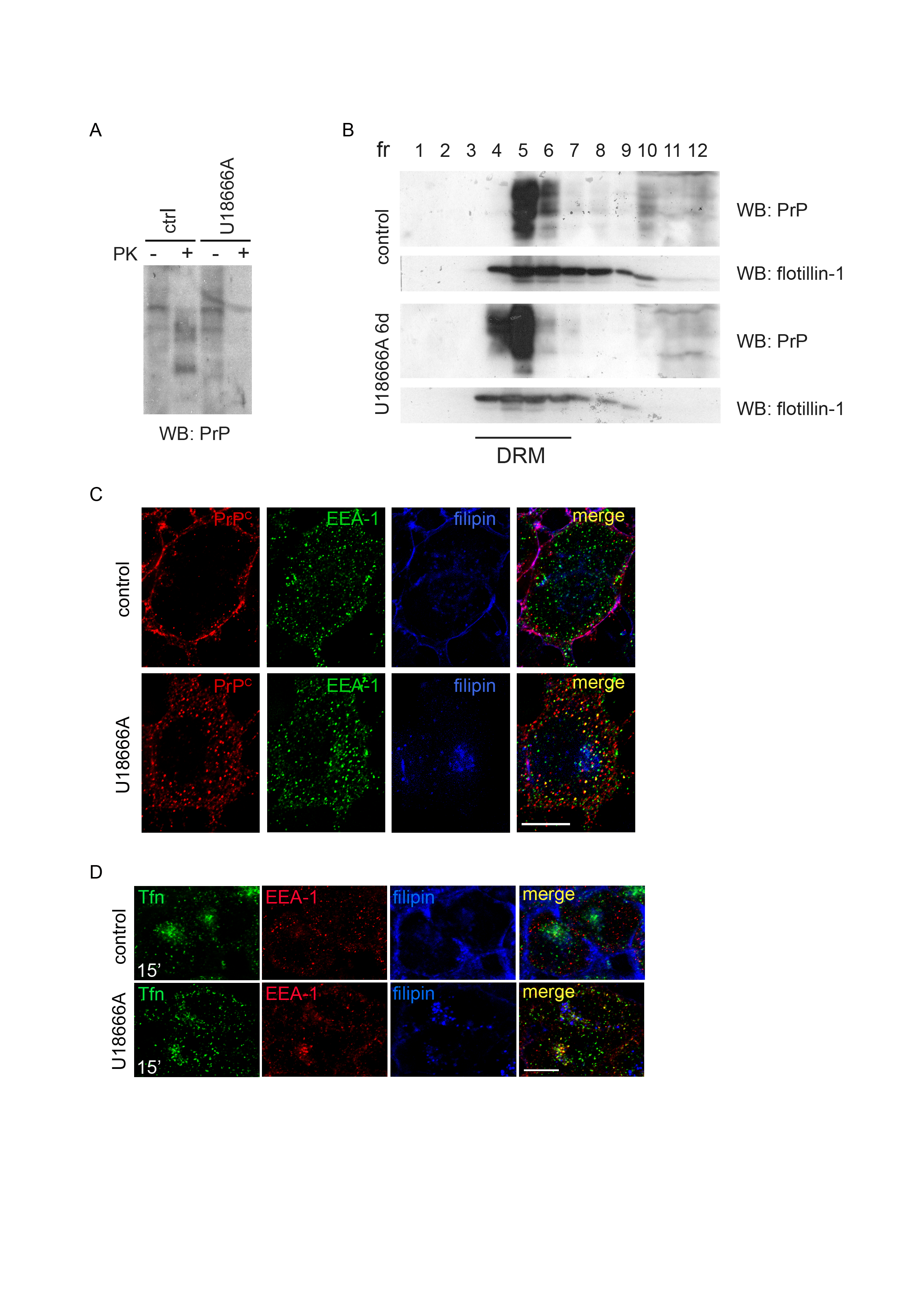

Supplement: Figure S3 — U18666A treatment reduces PrPSc levels in ScN2a cells and impairs trafficking of Tfn and PrP, without affecting PrPC distribution in detergent resistant domains (DRMs). (A) Control ScN2a cells and cells treated with 1 µM U18666A for 6 days were lysed and levels of total PrP or PrPSc were analyzed on western blot using SAF61 mAb. To reveal PrPSc lysates were incubated with 20 µg/ml of Proteinase K (PK). Note that no PrPSc could be observed in U18666A treated cells. (B) Lysates from control and U18666A treated ScN2a cells were applied on sucrose gradient and ultracentrifuged at 200000 g for 16 hr. Twelve fractions were collected and proteins were precipitated using 10% of trichloroacetic acid. PrP and flotillin-1 contents in each fraction were analyzed on western blot using SAF61 mAb and flotillin-1 mAb. Fractions 4–7 correspond to detergent resistant membranes (DRM) based on distribution of flotillin-1, which is mainly present in DRMs. In ScN2a cells PrP is distributed in DRMs and U18666A treatment does not change its distribution. (C) Steady-state localization of PrPC was analyzed in control (ctrl) and U18666A-treated cells after 6 days of treatment. The effect of the treatment was assessed by cholesterol accumulation in LE based on filipin staining shown in blue. Yellow colour represents colocalization between PrPC and EEA-1. Note that in control condition PrPC and cholesterol were exclusively localized at the cell surface, while upon U18666A treatment, cholesterol was redistributed to LE and PrPC was enriched in EEA-1 positive EE. (D) Alexa 488-Tfn was internalized for 15 min and its subcellular distribution was analyzed in control and U18666A-treated cells. Yellow colour represents colocalization between EEA-1 and Alexa 488-Tfn. Note that in U18666A-treated cells Alexa 488-Tfn is confined to EE and does not accumulate in the ERC. Scale bars 10 µm. (2.26 MB TIF) [file ppat.1000426.s003.tif]

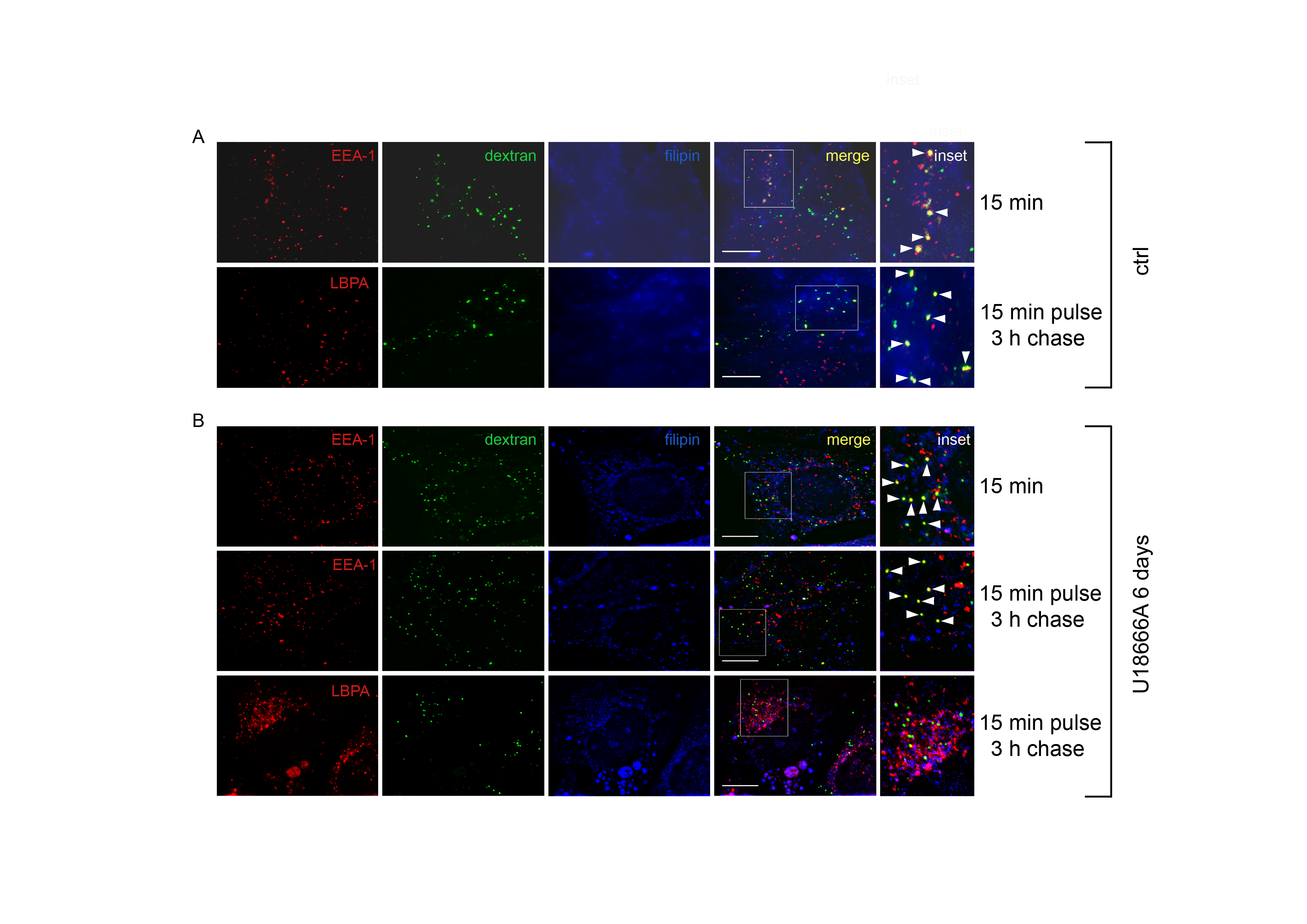

Supplement: Figure S4 — U18666A blocks EE to LE traffic in ScGT1 cells. (A) To study traffic from early (EE) to late endosomes (LE), control ScGT1 cells were either incubated with Alexa 488-dextran for 15 min and then fixed, permeabilized and immunolabeled for EEA-1, or additionally incubated in a dextran-free medium for 3 h and then fixed, permeabilized and immunolabeled for LBPA. Yellow colour and arrowheads indicate colocalization. In control cells a diffuse cholesterol distribution was revealed by fillipin staining. Inset represents magnification of the boxed area. Upon 15 min pulse dextran was internalized into EE and then was chased to LE upon subsequent incubation in dextran-free medium. (B) The experiment described in (A) was performed in ScGT1 cells treated with 5 µM U18666A. Characteristic cholesterol laden late endosomes were revealed with both filipin staining (blue) and immunolabeling for LBPA (red, lower panels). Magenta colour represents colocalization between filipin and LBPA in lower panels. Yellow colour and arrowheads indicate colocalization between dextran and EEA-1. Treated cells were able to internalize dextran into EE upon 15 min pulse, but in contrast to control cells they were not able to deliver dextran to the LE after 3 hr chase period. Scale bars 10 µm. (2.47 MB TIF) [file ppat.1000426.s004.tif]

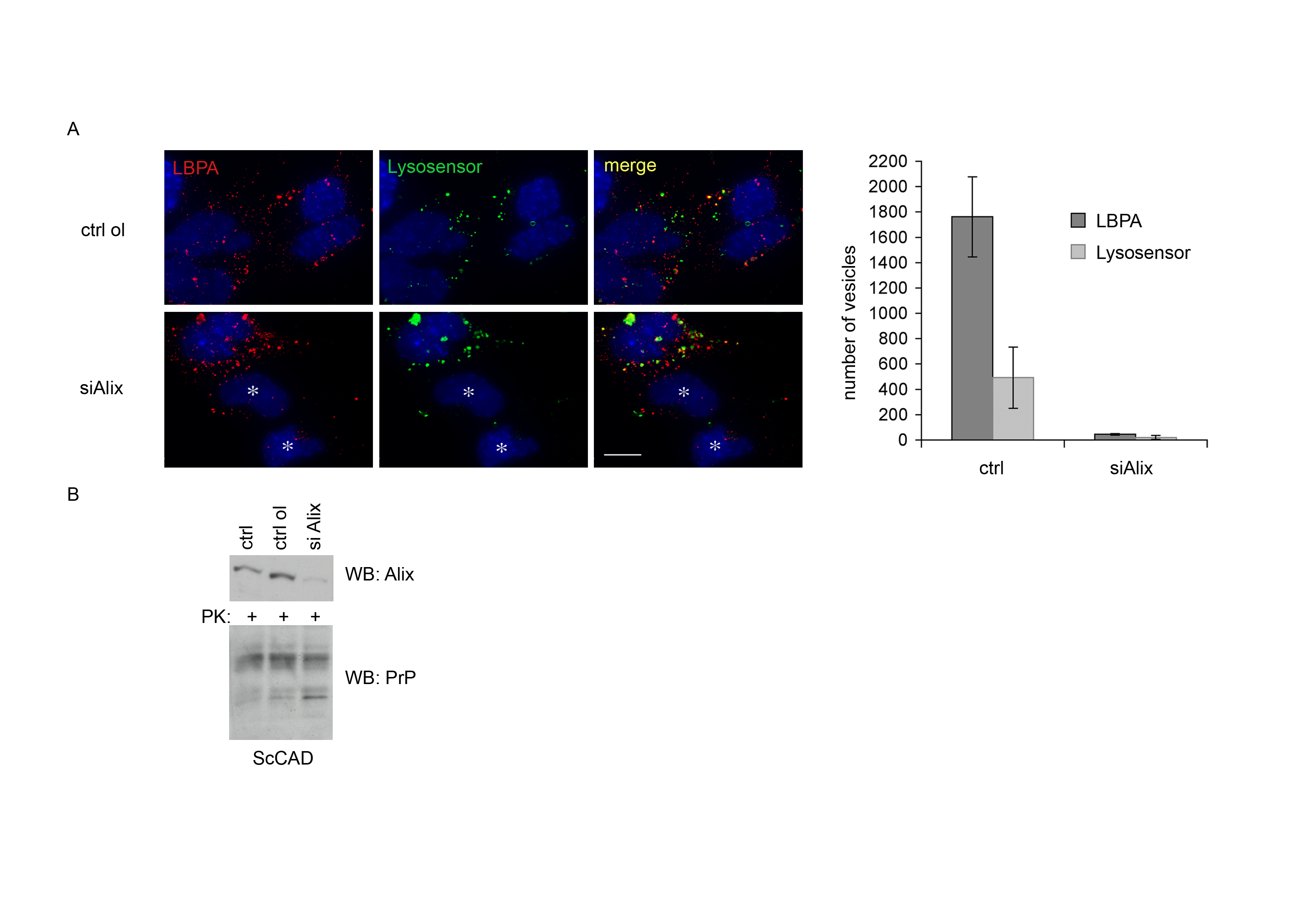

Supplement: Figure S5 — Alix downregulation in ScGT1 and ScCAD cells causes reduction in the number of late endosomes/lysosomes without affecting PrPSc levels. ScGT1 and ScCAD cells were transfected with either control oligo (ctrl ol) or RNAi against Alix (siAlix) during six days. (A) ScGT1 cells were let to internalize Lysosensor, which emits fluorescence only in acidic compartments followed by fixation, permeabilization and immunolabeling for LBPA. Scale bars 10 µm. The number of Lysosensor and LBPA positive vesicles was analyzed by Image J software. Results are presented as number of vesicles (mean±s.e.m) counted in 60 cells from 2 different experiments. Significant decrease in number of Lysosensor and LBPA positive vesicles was observed in Alix depleted ScGT1 cells when compared to control cells (p = 0,0011, t-test). (B) Alix and PrPSc levels were analysed by western blot in lysates from ScCAD cells transfected with Alix siRNA for 6 days. Levels of PrPSc were analyzed after the treatment with 20 µg/ml of PK using SAF61 mAb. PrPSc levels were not affected upon Alix downregulation. Similar results are obtained for ScGT1 cells (Figure 4). (1.26 MB TIF) [file ppat.1000426.s005.tif]

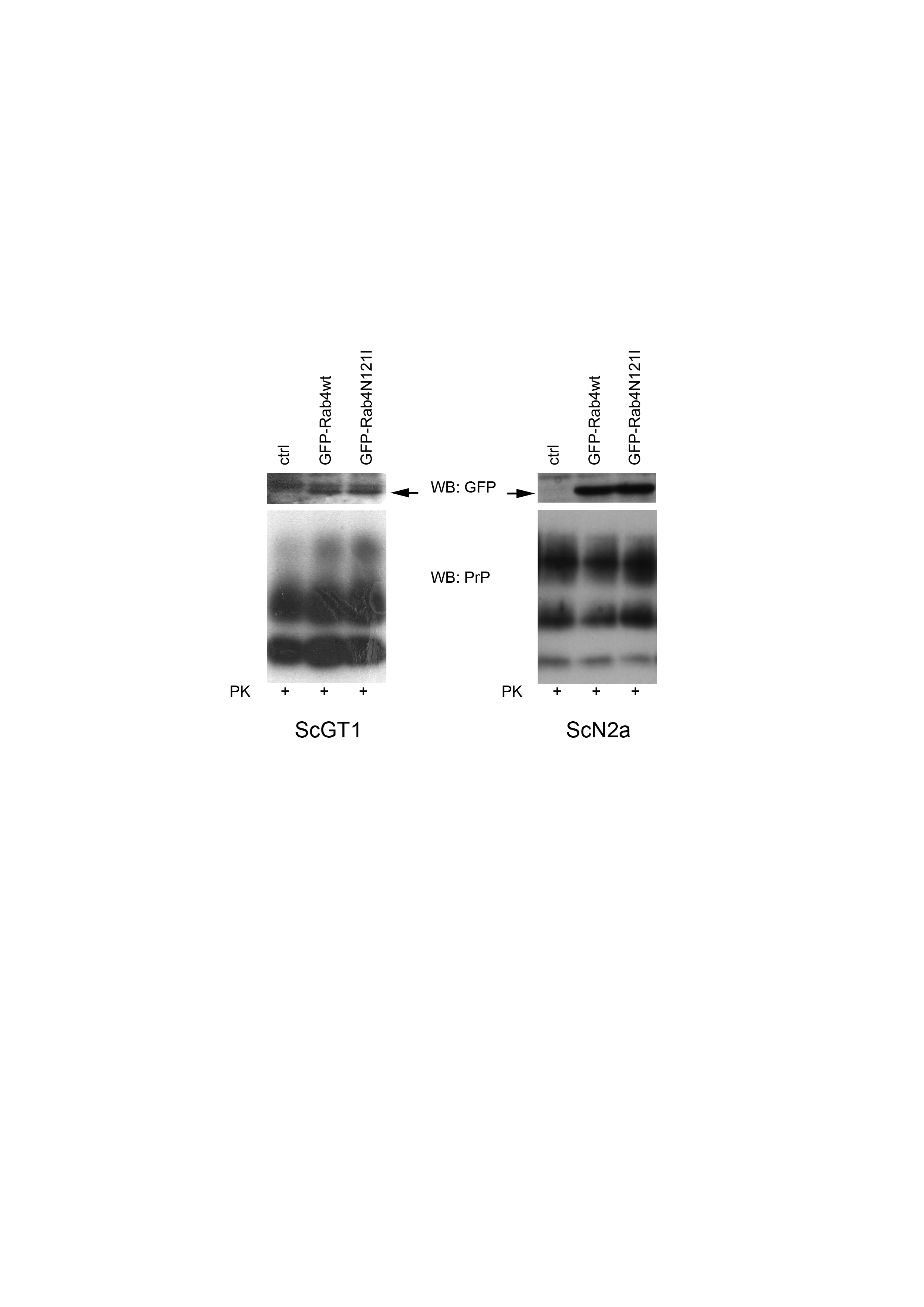

Supplement: Figure S6 — Expression of GFP-Rab4wt or GFP-Rab4N121I in ScGT1 and ScN2a cells does not influence PrPSc levels. Control ScGT1 and ScN2a cells and cells transfected with GFP-Rab4 constructs for 6 days were lysed and levels of GFP-Rab4 were analyzed on western blot using anti-GFP Abs. Bands corresponding to GFP-Rab4wt and GFP-Rab4N121I were marked by arrows. Levels of PrPSc were analyzed after the treatment with 20 µg/ml of PK using SAF61 mAb. (0.21 MB TIF) [file ppat.1000426.s006.tif]

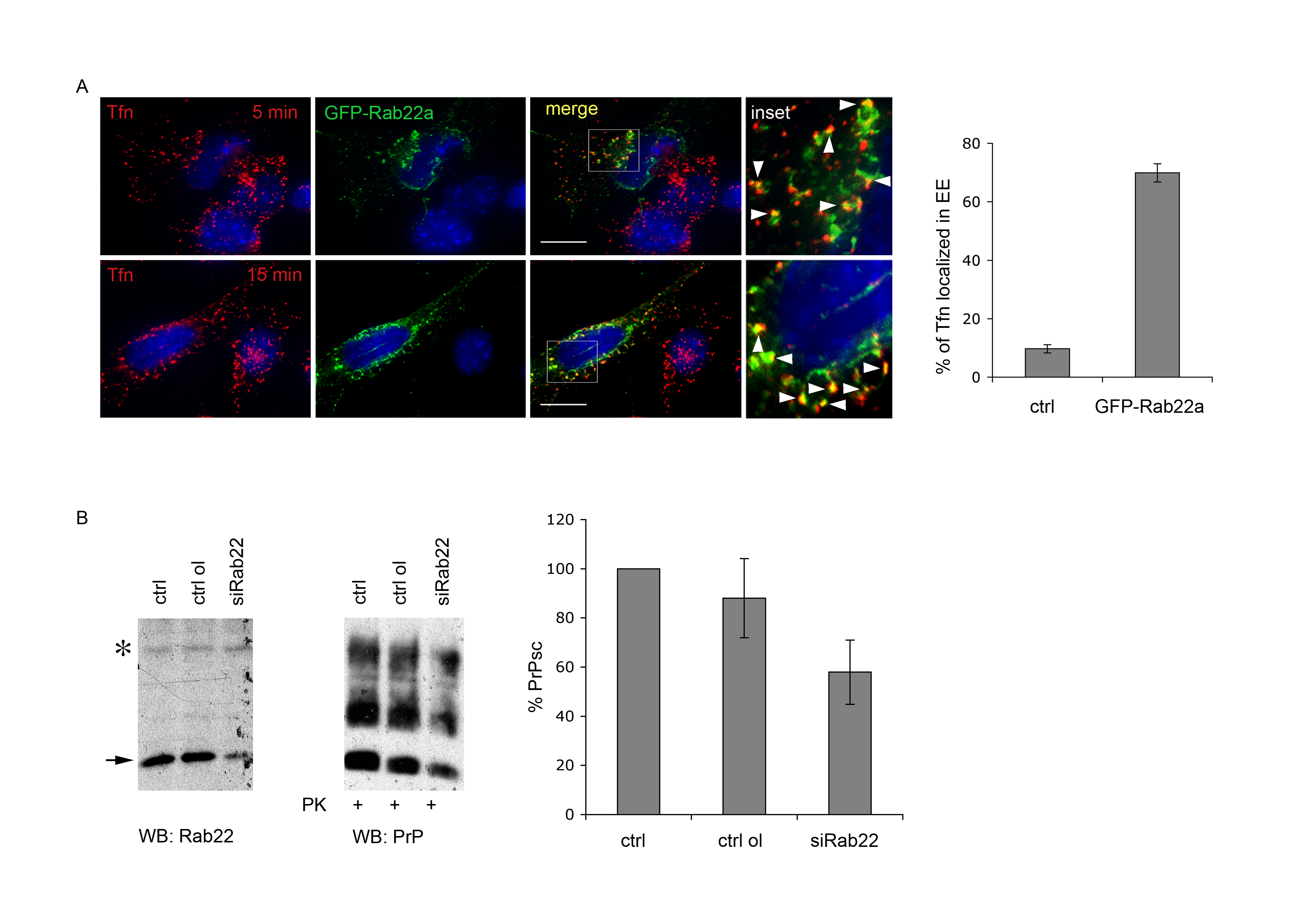

Supplement: Figure S7 — Rab22a regulates transferrin sorting to ERC and PrPSc production in ScGT1 cells. (A) Overexpression of GFP-Rab22a in ScGT1 cells inhibits Tfn sorting to ERC. ScGT1 cells were transfected with GFP-Rab22a and allowed to internalize Alexa 546-transferrin (Tfn) for 5 and 15 min. Yellow colour and arrowheads indicate colocalization. Scale bars 10 µm. The quantification results (mean±s.e.m, n = 36) are presented as % of total signal in red (Tfn) colocalizing with GFP-Rab22a (green). Increase of Tfn (p = 0,0099, t-test) in EE of GFP-Rab22a expressing cells was detected. Note that Tfn was not able to reach ERC after 15 min internalization. (B) Rab22a depletion reduces PrPSc level in ScGT1 cells. Rab22a and PrPSc levels were analyzed by western blot in lysates from untransfected cells (ctrl), or cells transfected with either control oligo (ctrl ol) or RNAi against Rab22a (siRab22) for 6 days. To reveal PrPSc the lysates were treated with 20 µg/ml of Proteinase K (PK) and SAF61 mAb was used on western blot. Around 60% downregulation in Rab22a level was observed in cells transfected with siRab22, 6 days post-transfection. The band corresponding to Rab22a is marked by an arrow. Asterisk indicates unspecific band as a control for equal loading. PrPSc levels were quantified and the results (mean±s.e.m, n = 3 experiments) are presented as % of PrPSc level in control, untransfected cells, which is considered as 100%. Around 50% reduction in PrPSc levels was observed in Rab22a depleted cells (p = 0,032, t-test). (1.97 MB TIF) [file ppat.1000426.s007.tif]

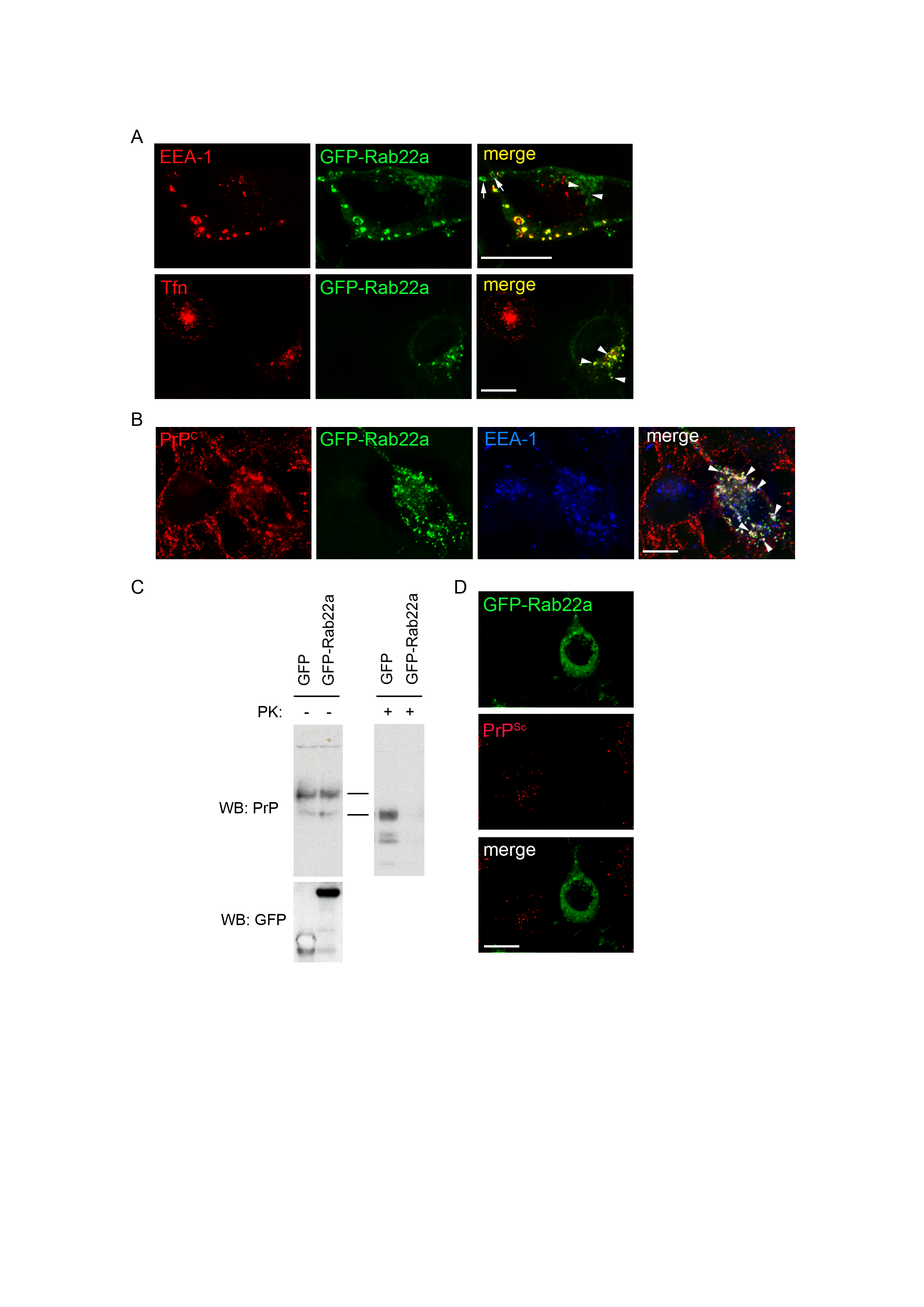

Supplement: Figure S8 — Overexpression of GFP-Rab22a affects cellular distribution of Tfn and PrPC and reduces PrPSc levels in ScCAD cells. (A) ScCAD cells transfected with GFP-Rab22a for 6 days were fixed and immunolabeled for EEA-1 (upper panels). In the parallel experiment (lower panels) transfected cells were let to internalize Alexa 546-Tfn for 15 min. Yellow colour represents colocalization between GFP-Rab22a and EEA-1 or GFP-Rab22a and Tfn. GFP-Rab22a was colocalizing with EEA-1 in EE, but it was also present in tubular structures (arrowheads) and EEA-1 negative vesicles (arrows). In contrast to control cells in GFP-Rab22a-expressing cells Tfn was not accumulating in the ERC, but was distributed instead in GFP-Rab22a positive compartment. (B) Cells transfected with GFP-Rab22a were fixed and immunolabeled for PrPC using Saf32 mAb and EEA-1 using anti-EEA-1 Ab. White colour and arrowheads represents colocalization between PrPC, GFP-Rab22a and EEA-1. (C) GFP and GFP-Rab22a in transfected ScGT1 cells were analyzed on western blot using anti-GFP Abs. Levels of PrPSc (PK+) and total PrP (PK−) were analyzed on western blot. (D) PrPSc was additionally analyzed by immunofluorescence in GFP-Rab22a transfected cells upon Gnd denaturation and immunostaining with POM-1 mAb. While PrPSc was revealed in control cells, no signal for PrPSc was observed by both western blot analysis and immunofluorescence in GFP-Rab22a-expressing cells. Scale bars 10 µm. (1.69 MB TIF) [file ppat.1000426.s008.tif]

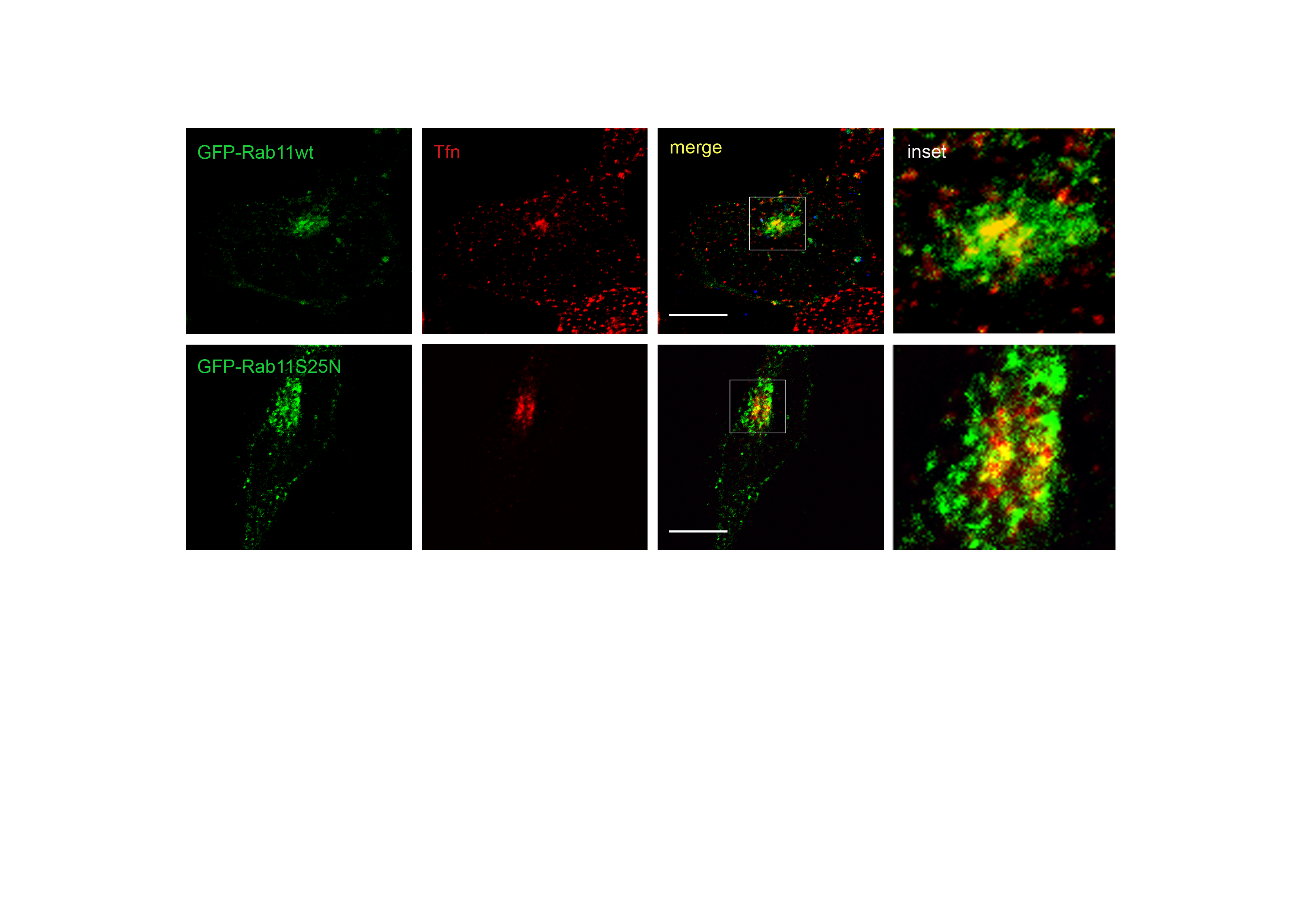

Supplement: Figure S9 — Rab11wt-GFP and Rab11S25N-GFP are partially localized in the ERC and in peripheral vesicles. ScGT1 cells were transfected with Rab11 constructs for 6 days. Alexa 546-transferrin was internalized for 15 min to label ERC. Yellow colour represents colocalization. Scale bars 10 µm. (1.69 MB TIF) [file ppat.1000426.s009.tif]
